# Predicting the impacts of land management for sustainable development on depression risk in a Ugandan case study

**DOI:** 10.1038/s41598-022-14976-3

**Published:** 2022-07-08

**Authors:** Thomas Pienkowski, Aidan Keane, Eugene Kinyanda, Caroline Asiimwe, E. J. Milner-Gulland

**Affiliations:** 1 Department of Zoology, University of Oxford, Oxford, OX1 3SZ UK; 2 School of GeoSciences, University of Edinburgh, Edinburgh, EH9 3FF UK; 3 Medical Research Council/Uganda Virus Research Institute and London School of Hygiene and Tropical Medicine Uganda Research Unit, 51-59 Nakiwogo Street, Entebbe, Uganda; 4 Budongo Conservation Field Station, Masindi, Uganda; 5 Jane Goodall Institute, Plot 26 Lugard Avenue, P.O. Box 462, Entebbe, Uganda

**Keywords:** Conservation biology, Ecosystem services, Psychology and behaviour, Sustainability, Psychiatric disorders, Depression

## Abstract

Agricultural intensification and expanding protected areas are proposed sustainable development approaches. But, their consequences for mental health are poorly understood. This study aims to predict how forest conservation and contract farming may alter resource access and depression risk in rural Uganda. Residents (*N* = 695) in 11 communities in Masindi District were asked about their expectations under land management scenarios using scenario-based interviews, household characteristics and depression symptoms. Over 80% of respondents presented with a ‘business-as-usual forest access’ scenario expected reduced access to forest income and food over the next decade; this number climbed above 90% among ‘restricted forest access’ scenario respondents. Over 99% of those presented with two land access scenarios (‘business-as-usual land access’ and ‘sugarcane expansion land access’) expected wealthy households to gain land but poorer families to lose it, threatening to increase poverty and food insecurity among small-scale farmers. Bayesian structural equation modelling suggested that depression severity was positively associated with food insecurity (0.20, 95% CI = 0.12–0.28) and economic poverty (0.11, 95% CI 0.02–0.19). Decision-makers should evaluate the mental health impacts of conservation and agricultural approaches that restrict access to livelihood resources. Future research could explore opportunities to support mental health through sustainable use of nature.

## Introduction

The earth’s biological diversity is being lost at unprecedented rates, largely driven by habitat degradation, species overexploitation, pollution, invasive species introduction, and climate change^1^. At the same time, many people worldwide continue to face poverty, hunger, illness, and other threats to wellbeing^2^. Looking forward, therefore, humanity faces the joint challenge of enhancing human health and wellbeing while improving the state of nature^3^. Two intersecting global policy frameworks seek to address this joint challenge. The proposed post-2020 global biodiversity framework aims to catalyse “urgent action across society to put biodiversity on a path to recovery for the benefit of planet and people” as a step towards living in harmony with nature by 2050^4^. Simultaneously, the 17 United Nations 2030 Sustainable Development Goals (SDG) include ending poverty (SDG 1) and hunger (SDG 2) while reducing inequalities (SDG 10) and ensuring health for all (SDG 3)^5^.

How landscapes are managed will play a key role in addressing this joint challenge in the coming decades. For example, feeding the world’s population whilst protecting nature will require significant changes in how agroecological systems are organised^6^. Often, land management interventions change who has access to nature and its resources, with complex trade-offs and co-benefits in relation to multiple dimensions of wellbeing^7,8^. A core part of wellbeing is mental health, defined as the capacity of thoughts, emotions, and behaviours that enable people to realise their potential, cope with stresses, work productively, and contribute to their community^9,10^. Poor mental health is a leading threat to wellbeing and a major contributor to the global burden of disease^11^. For instance, mental disorders contributed around 35% of total years lived with disability in 2015, and over 260 million people were estimated to have had depression in 2017^12,13^. Yet, evidence of how land management can influence access to natural resources in ways that affect mental health is limited^14^. Several studies explore how climate change influences place-based relationships and livelihoods in ways that may affect mental health (e.g.^15–17^). One study explored how declining Atlantic Cod fisheries quotas led to a loss of livelihoods, which appeared to be a significant and chronic source of distress among affected fishers^18^. However, the authors are unaware of any studies on how landscape management changes access to resources that affect livelihood-related determinants of mental illness. As one of the first of its kind, this study seeks to help address this gap.

This limited understanding of how land management may indirectly influence mental health (through changes in access to nature) has several implications. First, there may be hidden trade-offs between mental health and other sustainable development targets that are poorly accounted for when designing land management policy. For instance, some land management approaches that seek to protect nature (SDG 15) could alter access to natural resources in ways that undermine progress toward mental health targets (SDG Target 3.4)^14^. Second, understanding these connections may reveal land management approaches that simultaneously contribute to mental health and other sustainable development objectives. For example, given the strong links between poverty and mental illness^19^, land management policies that promote sustainable natural resource use could support mental health. Anticipating these trade-offs and co-benefits could help decision-makers choose land management approaches that promote mental health within a functional and diverse biosphere^20^.

This article aims to predict how land management for sustainable development could alter access to nature in ways that affect mental health. Specifically, this research explores how agricultural intensification and restrictive protected areas may increase depression risk through a Ugandan case study. The study encourages policymakers to anticipate how land management approaches could alter access to nature in ways that either undermine or support mental health goals.

### Nature’s contributions and mental illness

**Figure 1:**
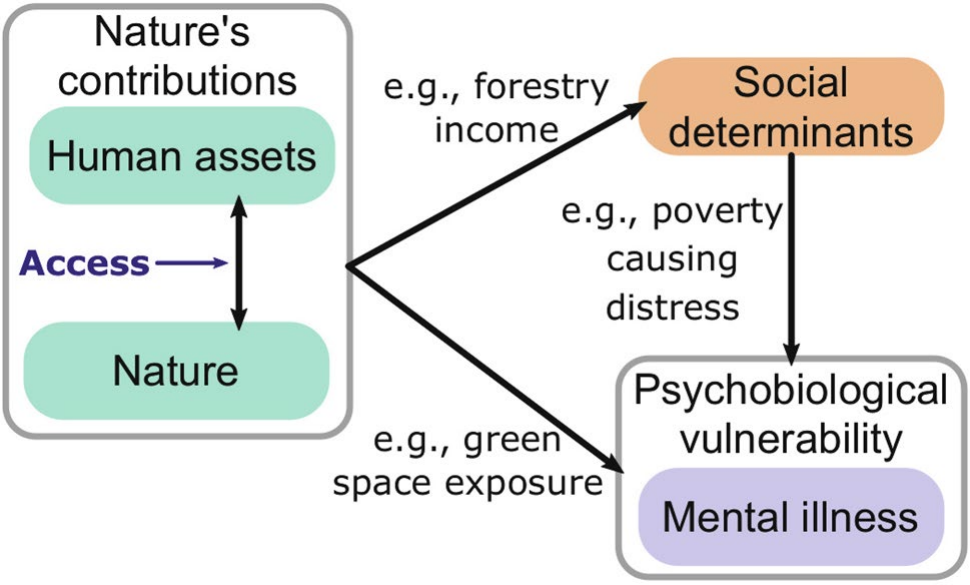
Illustrating how interactions between people and nature co-produce contributions that affect social determinants of mental illness, depending on an individual’s psychobiological vulnerabilities (adapted from Pienkowski et al.^14^). Access is one factor that mediates the interaction between people and nature.

The following illustrates how access to nature can influence social determinants of mental health, drawing on three bodies of evidence (Fig. 1). First, mental illness is defined as a disturbance of “thought, emotion, behaviour, and relationships with others that lead to substantial suffering and functional impairment” in major life activities^10^. Much of the burden of mental illness is attributed to common mental disorders, including depression, anxiety, and post-traumatic stress. Multiple factors influence an individual’s risk of mental illness, including the interaction of psychobiological vulnerabilities and external stressors^10^. These stressors can emerge from the economic, social, cultural, demographic, and environmental context of people’s lives, termed *social determinants* within public health literature^19^. For instance, poverty is a recognised social determinant of mental illness^19,21–23^. Many of these social determinants might be influenced by interactions with the natural world.

Second, nature refers to the natural world, with an emphasis on biodiversity^1^ (the variability of life on earth^24^). In this study, both land and forests are considered part of nature. The interaction of nature and human assets (like infrastructure, technology, or financial capital) co-produce food, energy sources, materials, medicine, and other contributions to human wellbeing^1^. These are termed *nature’s contributions* to people^25^. For example, wild foods harvested from forests support dietary diversity and good nutrition in many parts of the world^26,27^. Therefore, the interactions between human assets and nature may alter social determinants of mental illness. For instance, forest resources can contribute to food security, a known social determinant of common mental disorders^19,26^.

Finally, access has been defined as the “ability to benefit from things”^28^. An individual or group’s ability to access something can be determined by the rights, knowledge, authority, social relations, markets, and other factors available to them^28^. Access often mediates relationships between nature and people’s wellbeing^29–31^ and can be determined by formal and informal institutions and governance systems^25^. For instance, Thoms^32^ describes how elites within community forestry groups in Nepal restricted access to forests, harming the livelihoods of poorer households. Therefore, access is a crucial factor in determining who can co-produce and enjoy nature’s contributions^25^. Furthermore, multiple factors can change patterns of resource access, including land management policies and practices. For example, recent “land grabs” driven by agricultural modernisation, conservation, urbanisation, and other pressures have changed who has access to land in many parts of the world^33^. A wide range of actors can influence land management^34^. However, the current study is principally interested in how local and national governments control and incentivise land management practices. Specifically, the study explores how two landscape management approaches for sustainable development, agricultural intensification through contract farming and protected areas, alter patterns of access to land and forests.

Protected areas cover 15% of the world’s land area and range from categories that permit sustainable use to those strictly limiting public access^35^. Residents can face a wide range of benefits and costs from living next to protected areas, which can be highly contextual and differentiated between groups^36^. Protected areas are a critical approach within the post-2020 global biodiversity framework, with plans to nearly double their current extent by 2030^4,35^. This expansion is expected to affect the lives of hundreds of millions of people^37^, including changing patterns of access to nature. While the social impacts of this expansion might vary between contexts, there are concerns about its impact on Indigenous groups and other residents^38^.

Like protected areas, agriculture is a globally prevalent land use, covering 37% of the world’s land surface^39^. Contract farming has been promoted as a tool for sustainable development, offering the potential to increase food production, agricultural incomes, and employment while limiting nature loss^40^. Contract farming is where a processor agrees to purchase agricultural commodities from smallholder farmers under contract. Contracts can reduce farmers’ uncertainty about returns on investment and the transaction cost of finding buyers while improving access to inputs, finance, and extension services^41^. However, the distribution of benefits and costs of contract farming partly depends on who can engage in it and how it changes access to resources like land^42^. For instance, the expansion of contract farming had complex and differentiated social impacts in parts of Ghana, Kenya and Zambia but resulted in the consolidation of land by elites in several cases^43^. As such, the ability of contract farming to promote equitable sustainable development partly depends on how inclusive it is^40^.

The study focuses on area-based conservation and contract farming because they are globally prevalent, closely linked to rural livelihoods, and likely to play central roles in efforts to meet global sustainability targets^6^. Scenario-based interviews were used as predictive approaches to provide evidence of credible outcomes associated with potential intervention^44^. Scenario-based interviews can leverage local expertise to explore how and why people might behave under plausible future scenarios^44^. For example, Travers, et al.^44^ conducted scenario-based interviews with residents around a ‘reducing emissions from deforestation and forest degradation’ (REDD+) site in Cambodia. They found that collective payments to households or village development funds appeared more likely to reduce forest clearance than alternative conservation interventions. In addition, scenario-based interviews can be combined with other methods to provide nuanced predictions of future outcomes (e.g.^45^). We, therefore, combine interviews with a Bayesian structural equation model analysis, modelling pathways between nature use and depressive symptom severity. This combined approach illustrates how future land management may change access to forests and land (Component 1) in ways that influence social determinants of depression (Component 2) among communities around Budongo Forest in Masindi District, western Uganda. Component 1 explores how land management could influence access to land and forests, and in turn food and income, through hypothetical scenarios. Two research questions were asked in this Component:

- RQ1: What are the perceived impacts of restricted forest access on food security and economic poverty among forest-using households compared to business-as-usual (BAU) over the next decade?
- RQ2: What are the perceived impacts of an expansion of contract farming on land distribution, food security, and economic poverty compared to BAU over the next decade?

**Figure 2:**
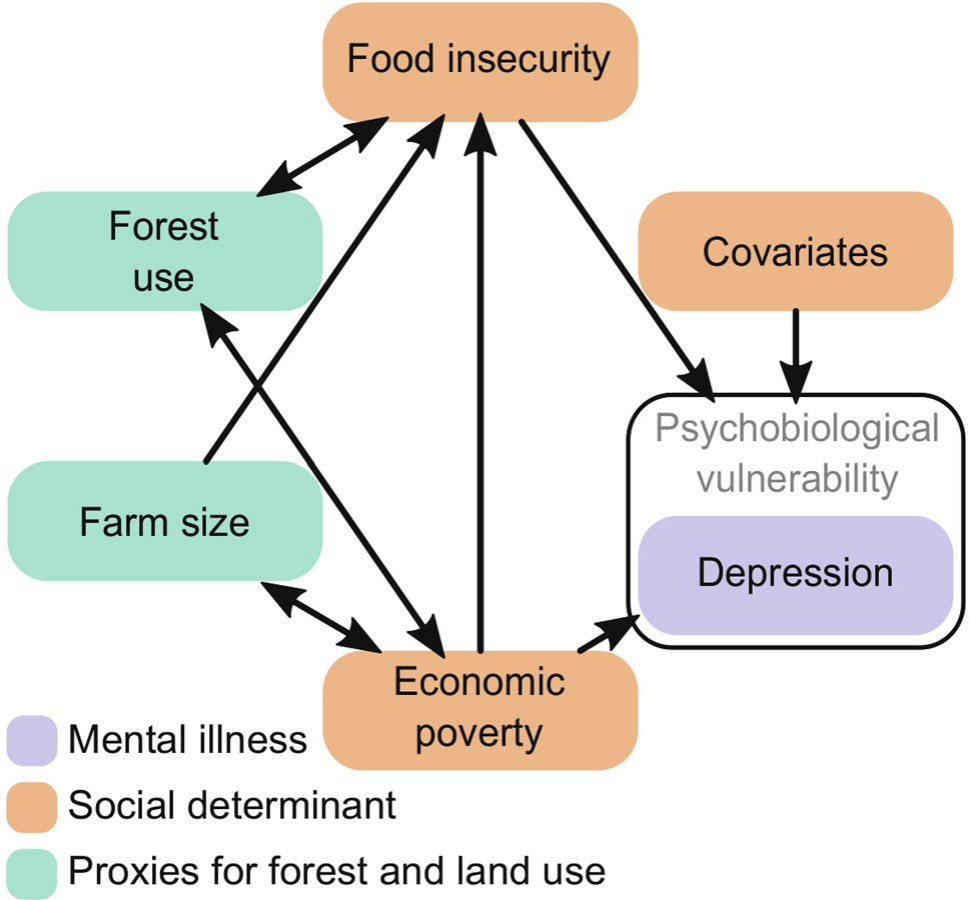
An illustration of the hypothesised links between farm sizes and forest use (proxies for nature’s contributions), food insecurity and depression (social determinants) and depression risk (mental illness) in the case study site. Single-headed arrows describe correlations, and bi-directional arrows describe co-variance.

Component 2 empirically models relationships between indicators of land and forest use, poverty and food security, and depression risk (Fig. 2). It is hypothesised that:

- H1: Forest use is positively associated with economic poverty and food insecurity.
- H2: Farm size is negatively associated with poverty and food insecurity.
- H3: Food insecurity and economic poverty are positively correlated with depressive symptom severity, controlling for covariates.

## Methods

### Brief description of the study area

**Figure 3:**
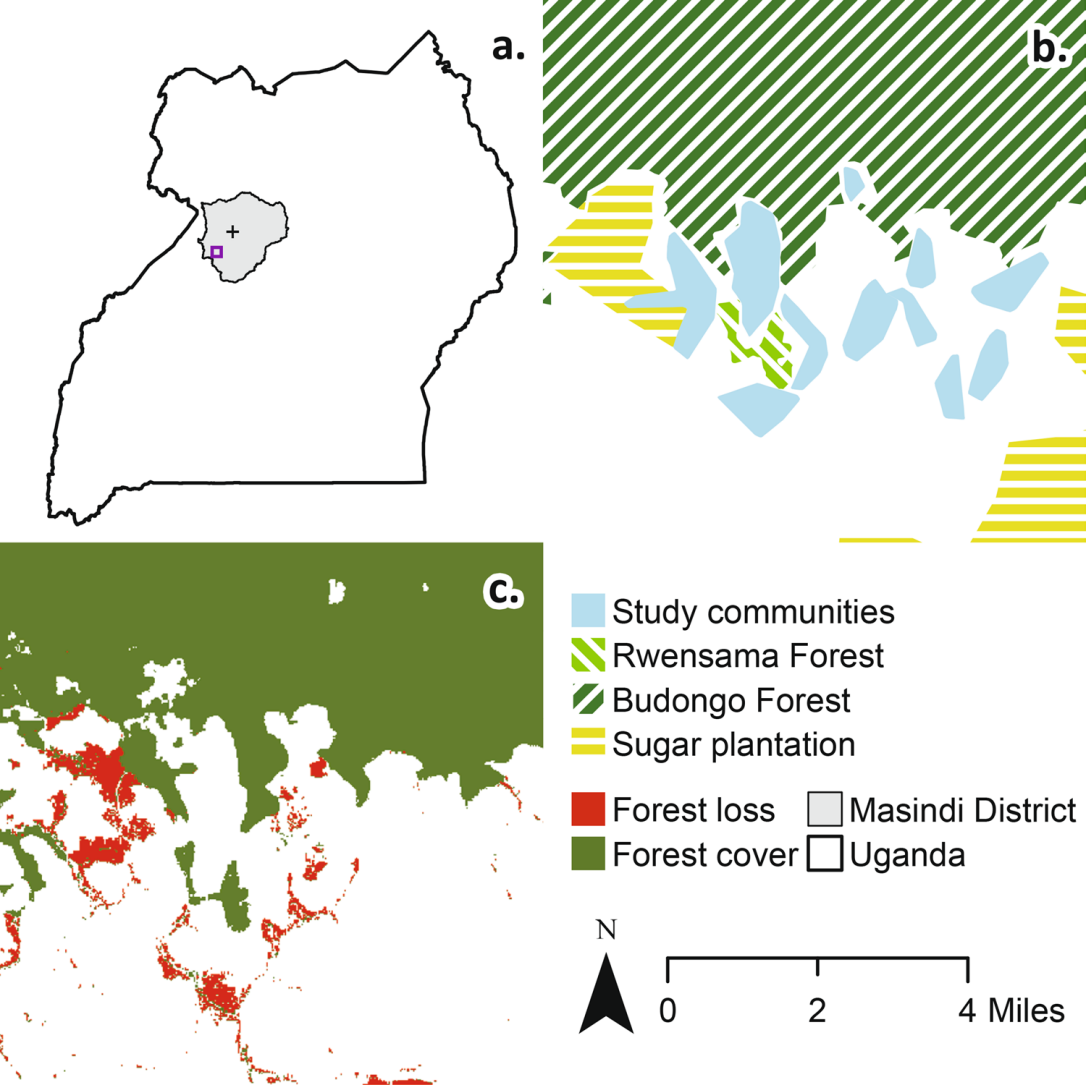
Maps describing the study area. Panel a. describes the location of the study site (purple box) in relation to Masindi Town (‘+’) within Masindi District, Uganda. Panel b. describes the study area, including the 11 study communities, the Budongo and Rwensama Forest Reserves, and the indicative location of large-scale intensive sugarcane estates (adapted from^35,47^). Panel c. describes forest loss between 2000 and 2016 and forest cover (> 75% tree cover) in 2016 (adapted from^48^).

The study area spanned 11 communities in Nyabyeya and Kabango parishes in Masindi District, Western Uganda (1.68° N, 31.54° E, Fig. 3). The study landscape is a mosaic of contract sugarcane and subsistence agriculture, bordering the Budongo and Rwensama Forest Reserves. The Ugandan government has adopted a wide range of reforms to modernise the country’s agricultural sector since 1986, leading to substantial growth in cash crop production, including sugarcane^46^. Contract sugarcane farming was promoted as a mechanism for inclusive development but has increased inequalities in several parts of the country^46^. Within the study area, expansion in sugarcane farming was perceived to have exacerbated inequitable land distributions, reportedly increasing incomes for wealthier landowners but worsening economic poverty, food insecurity, and consequent psychological distress among small-scale farmers^14^. This land redistribution appears to have occurred through several mechanisms, including voluntary selling or renting land to meet immediate needs and coercive or forced displacement^14^.

Forest reserves are essential to Uganda’s protected areas network, which covers 16% of the country’s land surface^35^. Budongo Forest Reserve, which neighbours the smaller Rwensama Forest Reserve, was gazetted by the British in the 1930s and is managed by the National Forestry Authority^49^. The forest is a source of illegal timber, charcoal, and wild meat and legally harvested medicines, firewood, plants and mushrooms for local residents^50^. These sources of forest food and income appear to alleviate stressors that cause psychological distress among poorer households^14^.

### Target population and sampling strategy

The target population includes male and female household heads (aged 18–60) of small-scale subsistence and contract farming households in the study area. This total population was estimated to include 2124 households, based on counts provided by community leaders. The research team systematically sampled this population by walking along all roads and paths in each community (identified with the help of community leaders), selecting every third household to be surveyed. Non-farming households were excluded from the systematic sample. Based on estimates provided by community leaders, the sampling strategy was expected to yield 708 respondents, representing approximately one-third of farming households. This sample size was deemed adequate in relation to the complexity of the statistical model. Respondents were surveyed in English, Kiswahili and Runyoro languages from March to May 2021. The survey was forward-translated by a professional translation service, then back-translated and refined with the help of five research assistants familiar with the local context and dialects over two weeks in March 2020. The survey was then piloted with 20 respondents in a neighbouring community outside the study area to ensure the questions were correctly interpreted (see Supplementary methods (SM) 1: Survey information and questions). Following this piloting, minor changes were made to the wording of questions and their translations to make them more easily interpreted.

### Component 1: Hypothetical landscape management scenarios

Many residents have witnessed changing forest and land access and management^14^. As such, the study participants were considered well-positioned to predict how hypothetical landscape management scenarios might affect their lives. Each respondent was randomly assigned a set of questions corresponding to one of four hypothetical scenarios (Table 1). One pair of scenarios contrasted a BAU scenario against one in which access to forests was restricted. The second pair of scenarios contrasted a BAU scenario against one in which government policies increased sugarcane prices, incentivising the expansion of contract farming. These scenarios were designed to reflect plausible land management approaches, informed by previous research in the study area^14^, that the Ugandan government might take to meet conservation and sustainable development targets. For instance, the *Ugandan National Development Plan 2020/21–2024/25* aims to raise farmers’ incomes by increasing land productivity by promoting contract farming, among other measures^51^.

**Table 1:** Four scenarios were presented to respondents, contrasting business-as-usual (BAU) scenarios against hypothetical changes in land management.

	Business-as-usual	Hypothetical intervention
Forest access	*BAU forest access*: “Some households in this community get food from the forest, like herbs, animals and mushrooms. In the next ten years, do you think households will get less food from the forest, more food from the forest, or will there be no change?” “Some households in this community get things from the forest to make money, like selling firewood, timber, and charcoal. In the next ten years, do you think households will get fewer things from the forest to make money, more things from the forest to make money, or will there be no change?”	*Restricted forest access*: “Some households in this community get food from the forest, like herbs, animals and mushrooms. I want you to imagine that people were not allowed to get anything from the forest, and there were more guards in the forest, over the next ten years. When you imagine this, do you think households would get less food from the forest, more food from the forest, or would there be no change?” “Some households in this community get things from the forest to make money, like selling firewood, timber, and charcoal. I want you to still imagine that people were not allowed to get anything from the forest, and there were more guards in the forest, over the next ten years. When you imagine this, do you think people would get fewer things from the forest to make money, more things from the forest to make money, or would there be no change?”
Land access	*BAU land access*: “Do you think there will be a change in who has land over the next ten years?”	*Sugarcane expansion land access*: “I want you to imagine that the price of sugarcane increased, and people wanted to grow more sugarcane over the next ten years. When you imagine this, do you think there would be a change in who has land over the next ten years?”

Respondents were asked about the expected consequences of each scenario on incomes and food availability through a combination of open- and closed-ended questions. These data were presented using descriptive statistics, and Pearson’s chi-square tests were used to explore differences between scenarios.

### Component 2: Structural equation modelling of relationships between respondent characteristics and mental health

Respondents were asked about their household, personal characteristics, and experiences of depressive symptoms. These data were analysed within a Bayesian structural equation model, as described below.

#### Study variables

The outcome variable within the statistical analysis was depressive symptom severity (Table 2). A modified version of the nine-item Patient Health Questionnaire (PHQ-9)^52^ was used to measure depressive symptom severity. The PHQ-9 is widely used and is a recommended depression screening tool in the *Diagnostic and Statistical Manual of Mental Disorders, Fifth Edition*^53^. It has been used in multiple studies in Uganda (e.g.^54,55^). One item related to suicidality appeared to be misinterpreted during piloting and was removed. This modified instrument, referred to as the PHQ-8, is increasingly used and has equivalent diagnostic accuracy with the PHQ-9^56^. The PHQ-8 asks respondents how many times they have experienced a given symptom in the past two weeks according to four response levels (with scoring from “Not at all” = 0 to “Nearly every day” = 3). Depression severity can be calculated by summing a respondent’s scores across items, with scores of 15–19 indicating moderately severe depressive symptoms and 20–24 indicating severe symptoms^57^. However, the PHQ-8 was used in this study to estimate latent depressive symptom severity (see *SM 2: Depression instrument*). Plausible values were extracted from a graded response model, implemented with ten imputed datasets (introduced below). Furthermore, “thinking too much” is a colloquial term associated with psychological distress in the study area and many parts of East Africa^58^. A one-item instrument asked if respondents experienced “thinking too much”, with the same response levels as the PHQ-8. This instrument was used to triangulate the current research results with a previous qualitative study^14^.

**Table 2:** The a priori hypothesised associations between exposure and outcome variables in the structural equation model and a description of the exposure variables.^a^

Outcome variable	Expected association	Exposure variable	Description of parent variable	Prior distribution^b^
Depression	(+)	Food insecurity	A latent variable derived from the Food Insecurity Experience Scale (FIES)^59,60^^c^	$N(0.25, 4)$
Depression	(+)	Economic poverty	An asset index adapted from Travers et al.^61^^c^	$N(0.25, 4)$
Food insecurity	(+) Co-variance	Forest use	A latent variable from an instrument designed to indicate forest use^c^	$B(\mathrm{12,11})$
Food insecurity	(−)	Farm size	A latent variable from an instrument designed to indicate relative farm size^c^	$N(-0.25, 4)$
Food insecurity	(+)	Economic poverty	As above^c^	$N(0.25, 4)$
Food insecurity	(−)	Distance to a forest reserve	Distance from the household to the edge of the nearest forest reserve^c^	$N(-0.25, 4)$
Economic poverty	(+) Co-variance	Forest use	As above^c^	$B(12, 11)$
Economic poverty	(−) Co-variance	Farm size	As above^c^	$B(11, 12)$
Depression	(+)	Age	The respondent’s age in years^c^	$N(0.25, 4)$
Depression	(+)	Gender	RL = male. The respondent’s gender	$N(0.25, 4)$
Depression	(−)	Education	RL = no education. The respondent's highest level of education	$N(-0.25, 4)$
Depression	(−)	Social support	A latent variable derived from a modified version of the Multidimensional Scale of Perceived Social Support (MSPSS)^62^^c^	$N(-0.25, 4)$
Depression	(+ /?)	Marital status	RL = married once or polygamous. Respondent's marital status	Divorced/widow/er: $N\left(0.25, 4\right)$ Never married: $N(0, 9)$
Depression	(−) Co-variance	Physical health	A single-item self-reported health question from the General Household Survey^63,64^^c^	$B(11, 12)$
Depression	(+)	Alcohol consumption	How many days a week does a respondent drink^c^	$N(0.25, 4)$
Depression	(+)	Smoking	If the respondent smokes every day	$N(0.25, 4)$
Depression	(?)	Community name	RL = Nyabyeya Trading Centre. The name of the community in which the respondent resides	$N(\mathrm{0,9})$

^a^Key: ‘+’ = positive association; ‘−’ = negative association; ‘?’ = uncertain direction of the association; RL = reference level, * N* = normal distribution (where the first argument is the mean and the second is the variance), *B* = beta distribution (with the arguments indicating the first and second shape parameters). All continuous variables are scaled and centred.

^b^See *SM 3: Prior probability* details for evidence supporting each prior.

^c^These variables are scaled and centred, and are thus presented in units of standard deviation.

The two focal social determinants of depression were food insecurity and economic poverty. Food security encompasses food sufficiency, nutrient adequacy, cultural acceptability, safety, certainty, and stability^65^. The study focused on food sufficiency, experienced as a continuum from worrying about not having enough to eat to reducing food consumption^59^. The Food Insecurity Experience Scale (FIES) developed by the FAO Voices of the Hungry project was used. The FIES is simple and rapid to use, appears valid across cultural and socioeconomic contexts, and has been used in studies in Uganda^65,66^. The FIES was adjusted, asking respondents to consider their experiences over a three-month timeframe, considered most relevant in the study. The FIES was used to estimate latent food insecurity in ten sets of plausible values, using a two-parameter logistic item response model (see *SM 4: FIES instrument*).

Here, economic poverty means inadequate incomes and wealth, considered core aspects of material poverty^67^. Asset ownership is often used to indicate material poverty^68^. An asset index was developed based on a survey created by other researchers during a study conducted in a nearby area in 2015, which was piloted and adapted in March 2019^61^. After data collection, ten of the 31 assets were excluded because they were either (a) very uncommon, (b) dependent on community-level access to utilities, or (c) appeared to depend on a respondent’s livelihood strategy (see *SM 5: Asset index*). Logistic principal component analysis was conducted with the remaining items. The first component scores were extracted, scaled and centred, and treated as a proxy for economic poverty.

The focal proxies for nature use were farm size and forest use. These were used as proxies because it was not feasible to directly measure interactions between residents and nature. Residents of the study site use a wide range of legally and illegally harvested forest resources^50^. Legally harvested resources included firewood, medicines, and some wild foods such as mushrooms. Illegal forest resources included wild meat, timber, and wood for charcoal production. Given the sensitivity around direct questioning of illegal behaviours, an instrument with seven Likert-scaled items (with five response levels ranging from “disagree a lot” to “agree a lot”) was designed to estimate forest use, also treated as a latent variable. These items included non-specific (focusing on the role of the forest in general) and specific (relating to household reliance on forest-related food and income) questions. This instrument was used to estimate latent forest use, following the steps described for estimating latent depression, with the extraction of ten sets of plausible values (see *SM 6: Forest instrument*).

Land boundaries and ownership are sensitive topics, and farms can be far from households where the surveys were conducted, so farm sizes were not physically measured. Instead, an instrument with six Likert-scaled items (with five response levels ranging from “disagree a lot” to “agree a lot”) was designed to estimate relative farm size. For example, one of the statements read, “Your household's farm is smaller than most others in this community.” This instrument was used to estimate latent relative farm size, following the steps described above for estimating latent depression, before extracting ten sets of plausible values (see *SM 7: Land instrument*).

The analysis also included several covariates identified during the prior qualitative study at the same site and by reviewing relevant literature (Table 2, see *SM 8: Social support instrument*). Psychobiological vulnerability can moderate the links between social determinants and mental illness but was assumed to vary randomly in the population in relation to the exposure variable and was not measured.

### Statistical analysis

Within the sample, 0.2% of the data were missing. These data were assumed to be missing at random (see *SM 9: Patterns of missing data*) and were substituted through multivariate imputation by chained equations. Ten datasets were created containing imputed values (see *SM 10: MICE*).

A Bayesian structural equation model was fit for each of the imputed datasets. The analysis was performed in the Stan computational framework (accessed using the ‘blavaan’ package^69^). The model's structure is based on the results of previous qualitative research in the study area (corresponding to Fig. 2). Moderately informative priors were chosen when there was evidence of an expected direction of effect. For instance, substantial evidence suggests positive associations between food insecurity and depression risk^19,70,71^. Weakly informative priors were used where there was little prior evidence of an expected direction of effect.

Using a seed value of 4343, the model was run for 4000 burn-in and 4000 post-burn-in iterations (8000 total), with the posterior distribution estimated with the Markov Chain Monte Carlo sampler, across four Markov chains, following McElreath^72^. The models were evaluated according to the ten steps described in the When to worry and how to Avoid the Misuse of Bayesian Statistics Checklist (WAMBS)-Checklist (see *SM 11: Model diagnostics*)^73^. Finally, the model results associated with the ten imputed datasets were pooled by combining the posterior distributions. Point estimates are the median of the posterior distribution.

Several post hoc supplementary analyses were implemented. First, the association between “thinking too much” and latent depressive symptom severity was modelled using a Bayesian ordinal regression with weakly informative priors (see *SM 12: Supplementary analysis 1*). Second, the primary analysis described above was repeated, replacing depressive symptom severity with “thinking too much” (treated as a continuous variable, see *SM 13: Supplementary analysis 2*).

### Ethical approval

Ethical approval was granted by the Uganda National Council of Science and Technology (Ref. SS6007) of the Government of Uganda and an Ethical Review Board at the University of Oxford, United Kingdom (Ref. R63458/RE002). All methods were carried out in accordance with relevant guidelines and regulations. Informed consent was obtained from all subjects.

## Results

The survey was completed by 695 respondents from 11 communities (Table 3). Of these, 11.2% reported moderately severe depressive symptoms (PHQ-8 scores = [15, 19]) and 4.6% reported severe symptoms (PHQ-8 scores = [20, 24]). Furthermore, reported experiences of “thinking too much” were positively associated with PHQ-8 scores (log odds = 1.27, 95% CI = 1.22–1.31). For instance, someone with a PHQ-8 score of 20 was 2.38 times more likely to report “thinking too much” nearly every day than someone with a score of 10 (see *SM 12: Supplementary analysis 1*).

**Table 3:** Overall and gender-differentiated respondent characteristics.

Characteristic	Overall N = 695	Female N = 414	Male N = 281
PHQ-8 score	9.7 (4.9)	10.1 (5.0)	9.1 (4.8)
**Strong thoughts?**			
Not at all	96 (14%)	63 (15%)	33 (12%)
Few days	241 (35%)	132 (32%)	109 (39%)
More than half the days	97 (14%)	54 (13%)	43 (15%)
Nearly every day	261 (38%)	165 (40%)	96 (34%)
FIES score	4.9 (2.5)	5.2 (2.5)	4.6 (2.5)
Age	35.6 (11.4)	34.8 (11.1)	36.8 (11.8)
**Education**			
No education	63 (9.1%)	58 (14%)	5 (1.8%)
Primary	465 (67%)	282 (68%)	183 (65%)
Secondary	144 (21%)	67 (16%)	77 (27%)
Beyond Secondary	23 (3.3%)	7 (1.7%)	16 (5.7%)
**Marital status**			
Divorced or widow/er	117 (17%)	95 (23%)	22 (7.8%)
Married/polygamous	532 (77%)	298 (72%)	234 (83%)
Never married	46 (6.6%)	21 (5.1%)	25 (8.9%)
**Health**			
Very bad	31 (4.5%)	19 (4.6%)	12 (4.3%)
Bad	75 (11%)	51 (12%)	24 (8.5%)
Fair	364 (52%)	208 (50%)	156 (56%)
Good	159 (23%)	97 (23%)	62 (22%)
Very good	66 (9.5%)	39 (9.4%)	27 (9.6%)
Alcohol consumption	0.5 (1.2)	0.3 (1.0)	0.7 (1.4)
**Daily smoker**			
No	619 (89%)	395 (95%)	224 (80%)
Yes	76 (11%)	19 (4.6%)	57 (20%)
Language			
English	96 (14%)	37 (8.9%)	59 (21%)
Kiswahili	502 (72%)	312 (75%)	190 (68%)
Runyoro	97 (14%)	65 (16%)	32 (11%)

Numeric variables are described by their mean (and standard deviations). Categorical data are described by their counts (and percentages). Key: PHQ-8 = eight-item Patient Health Questionnaire, FIES = Food Insecurity Experience Scale, Alcohol consumption = how many days a week a respondent consumes alcohol.

### Component 1: Effects of land management scenarios on access

**Figure 4:**
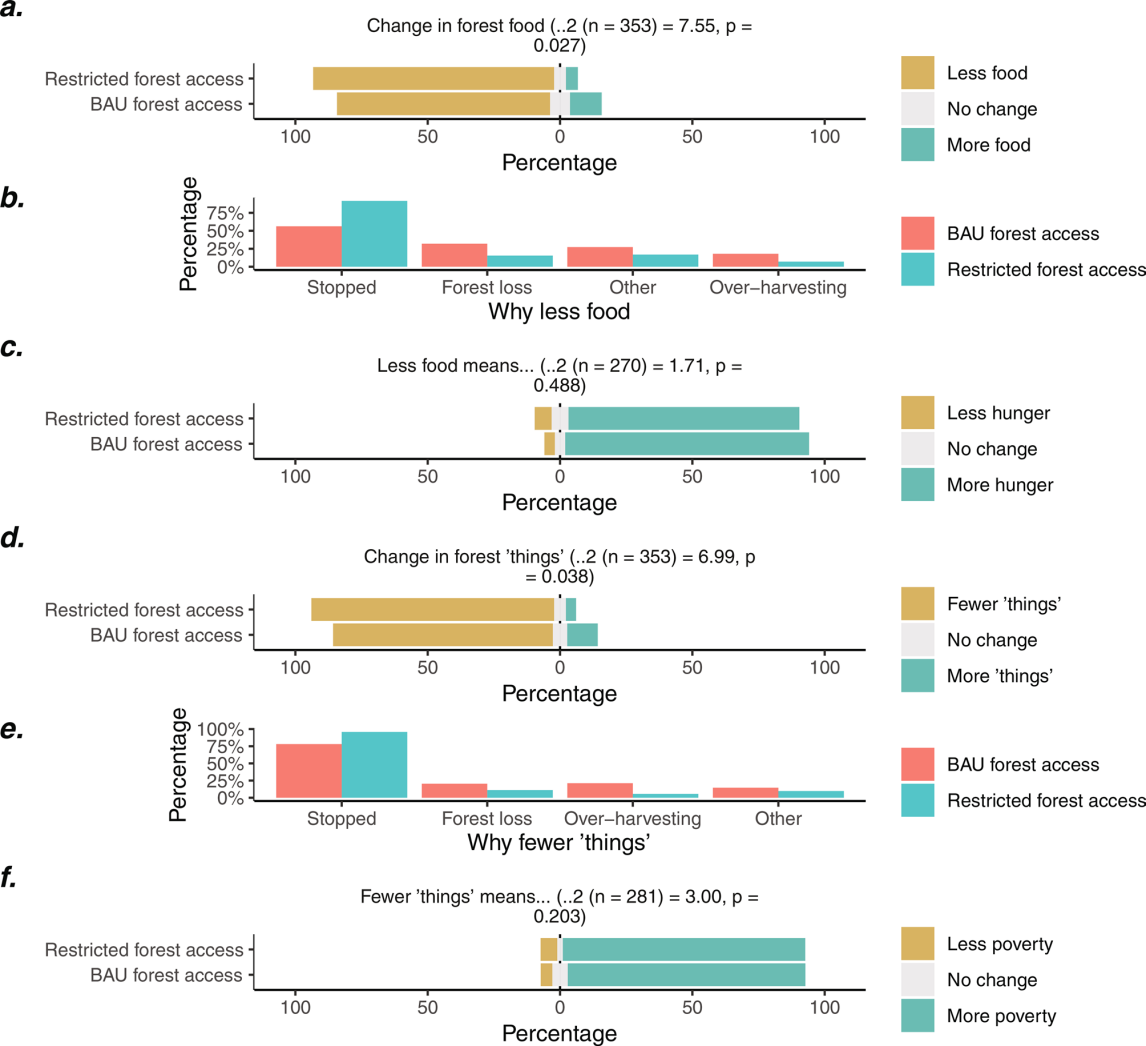
Scenario-based interview responses to two forest access scenarios. Panel a. describes expected changes in the amount of food from forests over the next decade, panel b. shows reported reasons for this decline, and panel c. illustrates expected consequences for household hunger (among forest users). Panel d. shows expected changes in the amount of income-generating ‘things’ from the forest, panel e. describes the reported reasons for this decline, and panel f. displays the expected consequences for household poverty (among forest users). Key: BAU = business-as-usual.

In total, 353 respondents were asked about either the ‘BAU forest access’ (188) or ‘restricted forest access’ (165) scenarios. Most of these respondents felt households would get less food (Fig. 4a.) and fewer income-generating ‘things’ (Fig. 4d) from the forest over the next ten years. The expected decline in food and ‘things’ were significantly higher among ‘restricted forest access’ scenario respondents. Many of those expecting declines indicated that this would be because residents would be stopped from accessing the forest (Fig. 4b,e), particularly in the ‘restricted forest access’ scenario. The majority believed that households who got less food and fewer ‘things’ from the forest would experience greater food insecurity (Fig. 4c) and poverty (Fig. 4f), with no significant difference between scenarios.

**Figure 5:**
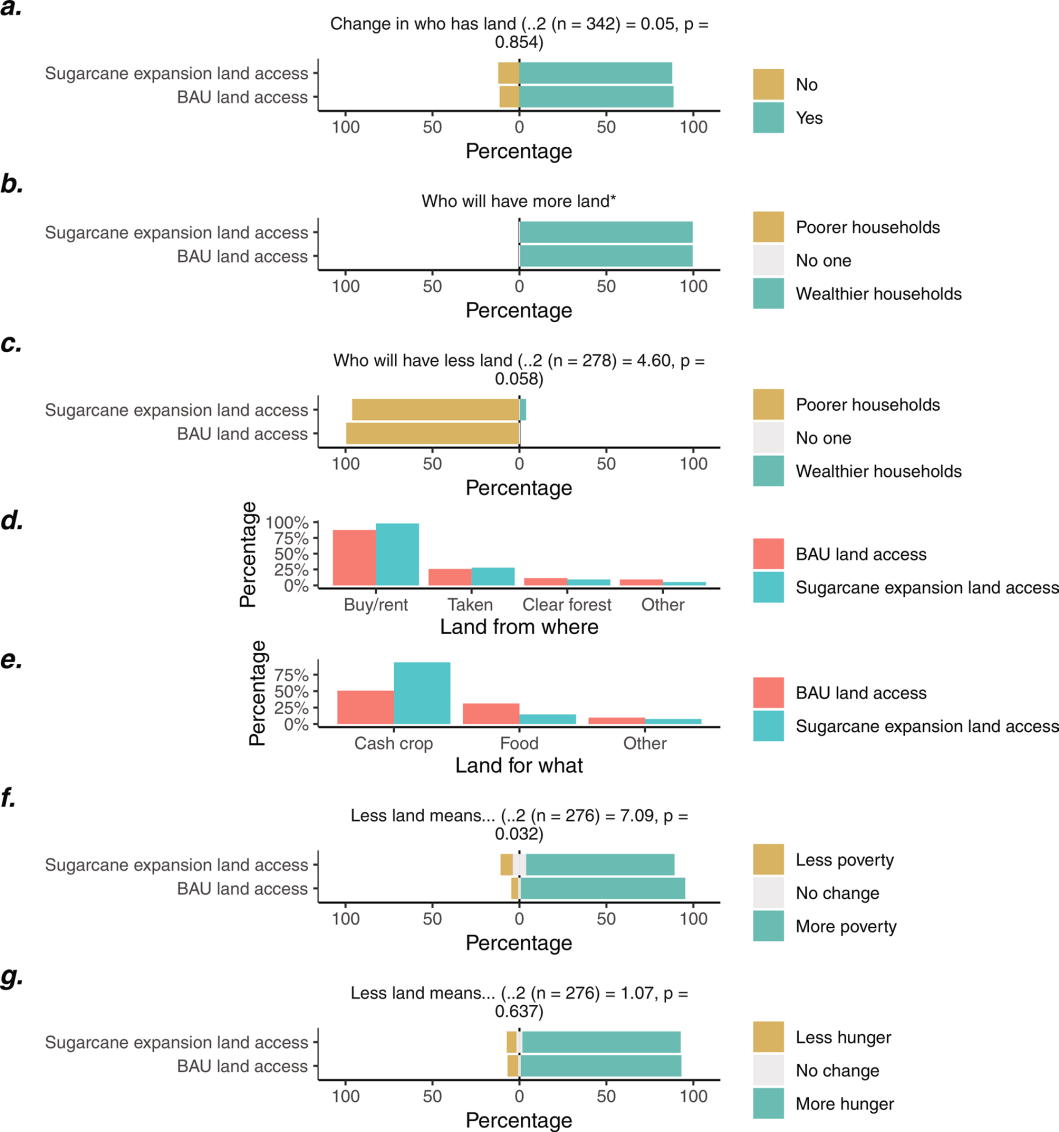
Scenario-based interview responses to two land access scenarios. Panel (**a**) describes the proportion expecting changes in who has land over the next ten years, and panel (**b**) and (**c**) illustrates who is predicted to gain and lose land, respectively (among those expecting a change). Panel (**d**) describes where this land is expected to come from, and panel e. displays what this land might be used for. Panel (**f** and **g**) illustrate the expected impacts of losing land on household poverty and hunger, respectively. *Where the chi-square test could not be formed because no respondents expected poorer households to gain land. Key: BAU = business-as-usual.

The other half of the respondents (342) respondents were asked to consider the ‘BAU land access’ (163) and ‘sugarcane expansion land access’ (179) scenarios. Most of these respondents expected a change in who has land over the next ten years, with no significant difference in responses between the two scenarios (Fig. 5a). Among those expecting a change, almost all said wealthier households would gain land, and poorer households would lose it, with no significant difference between scenarios (Fig. 5b,c). While many indicated that households would buy or rent this land, a sizable proportion also believed this land would be “taken” from others (Fig. 5d). Many expecting a change in land distributions indicated that acquired land would be used for cash crops, particularly in the ‘sugarcane expansion land access’ scenario (Fig. 5e). In both scenarios, many indicated that a decline in a household’s land would lead to an increase in poverty (Fig. 5f) and hunger (Fig. 5g), although poverty increases were slightly lower in the ‘sugarcane expansion land access’ scenario.

### Component 2: The relationship between forest use, farm size and social determinants of depression

**Figure 6:**
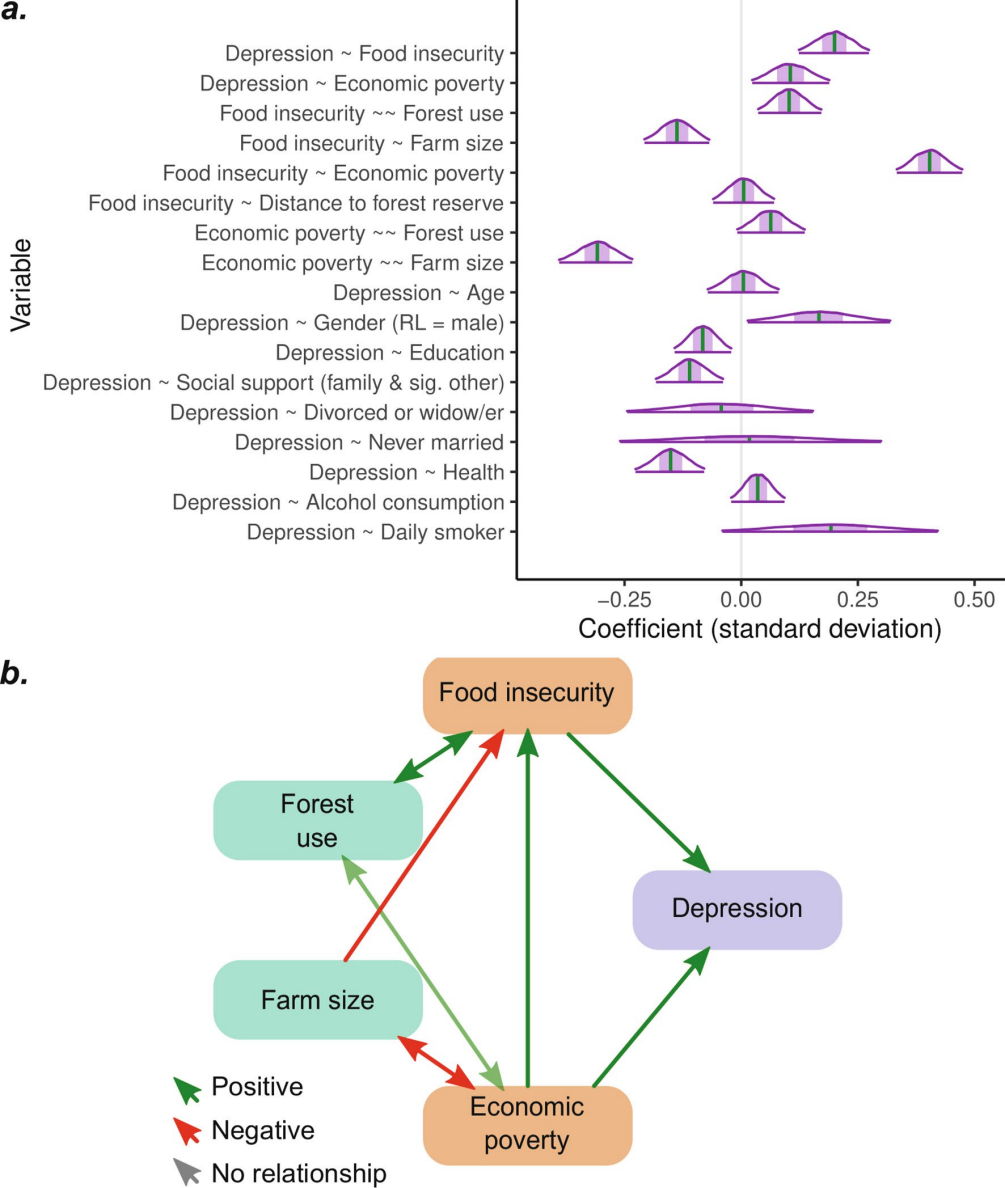
Bayesian structural equation modelling results. Panel (**a**) describes the coefficient estimates from the Bayesian structural equation model using data from 695 respondents. The vertical green line represents the point estimate (median of the posterior distribution), the dark purple line represents the 95% credibility interval, and the shaded area represents the 50% credibility interval. The estimated associations between depressive symptom severity and the community dummy variables are not shown. Coefficient estimates are presented in standard deviations. Panel (**b**) illustrates the direction of association between each variable (excluding covariates). The semi-opaque line indicates a marginally statistically uncertain association (as shown in Panel (**a**). Key: ‘ ~ ’ = regression, ' ~  ~ ' = co-variance, ‘RL’ = reference level.

Within the statistical analysis, forest use was positively associated with food insecurity and (with less statistical certainty) economic poverty (Fig. 6a). Conversely, farm size was negatively associated with economic poverty and food insecurity. Furthermore, food insecurity and economic poverty were positively associated with depressive symptom severity.

The analysis supported many of the hypothesised associations between depressive symptom severity and the covariates. For instance, men and those with higher education, greater social support, and better health reported lower depressive symptom severity. Additionally, the analysis was repeated with the software’s default weakly informative priors, the results of which were effectively the same as those presented here (see *SM 11: Model diagnostics*).

When looking at the total effects, those with one standard deviation (s.d.) higher forest use than the mean had an estimated 0.03 (95% CI = 0.00–0.05, Fig. 6b) s.d. higher depressive symptom severity. Similarly, those with one s.d. larger farm sizes than the mean reported 0.08 (95% CI = 0.11–0.05) s.d. lower depression severity. In other words, forest users were at a slightly greater risk of depression, but those with larger farms were at a slightly lower risk.

The analysis was repeated, substituting latent depressive symptom severity with an instrument asking about “thinking too much”. The results of this supplementary analysis were largely consistent with those presented above, although with some differences associated with the covariates (see *SM 13: Supplementary analysis 2*).

## Discussion

Landscape management can often change patterns of access to nature, with complex outcomes for human wellbeing^7,8^. Yet, the authors are unaware of any studies predicting how changing access to land and forest resources influence social determinants of mental illness. The current study helps address this gap, describing the expected consequences of changes in landscape management on social determinants of depression in a Global South case study. The following summarises the results in relation to the original research questions and hypotheses, discusses key study considerations, and then situates the results within a wider policy context.

Respondents expected that restricted forest access (in both forest scenarios) would increase food insecurity and economic poverty among forest-using households (RQ1). Moreover, the modelling results suggested that forest use was positively associated with food insecurity and, to a lesser extent, economic poverty (H1). Food insecurity and poverty appeared to be strong social determinants of depressive symptom severity (H3). Combined, these results suggest that conservation approaches that restrict forest access may exacerbate social determinants of depression among forest users. These results corroborate other studies in several ways. Studies have found that access to multi-use protected areas can sometimes support residents’ livelihood and food security^36^. For example, Naidoo, et al.^74^ synthesised data from over 60,000 households in 34 developing countries, finding that children under five living near multi-use protected areas with tourism had higher height-for-age scores and were less likely to be stunted than comparators far from protected areas. However, strictly protected areas that restrict access to nature may increase economic poverty and food insecurity in some cases^36^. For instance, Golden et al.^75^ estimated that removing access to wild meat would lead to a 29% increase in the number of children suffering from anaemia in rural north-eastern Madagascar. Furthermore, extensive evidence suggests that poverty and food insecurity are important social determinants of common mental disorders^19,21,71^. For example, Kinyanda, et al.^22^ found that indicators of poverty were associated with greater risks of depression among a nationally representative sample of 4660 Ugandans.

Furthermore, respondents also expected more inequitable land distribution, largely attributed to contract sugarcane farming, with those losing land at greater risk of food insecurity and economic poverty (RQ2). The statistical analysis suggested that farm size was negatively associated with poverty and food insecurity (H2). Together, these results suggest that agricultural approaches that restrict access to land may worsen social determinants of depression among small-scale farmers. These results align with other studies suggesting that contract farming can benefit participating households. For instance, Meemken and Bellemare^41^ evaluated nationally representative data from six countries, finding that contract farmers obtained higher incomes than non-contract farmers in some contexts. This study also corroborates research suggesting contract farming can increase inequality and worsen outcomes in some cases^42,43^. For example, in a case study around Kakira Sugar Works in Uganda, Martiniello^46^ found that the expansion of sugarcane contract farming marginalised poor smallholders. Moreover, contract farming may go hand-in-hand with other factors like global market exposure, indebtedness, and uncertain yields linked to high rates of suicide in some farming communities^76,77^.

There was relatively little difference within each scenario pair. This result could be because the hypothetical interventions resembled land management practices that were expected to occur regardless. For instance, residents who have witnessed the expansion of contract farming over the last two decades may expect this trend to continue. If this is the case, those who influence land management, such as the Ugandan government, should consider policies that actively divert from this trajectory, as discussed below.

### Study limitations

The study combined modelling of current epidemiological dynamics with expectations under different scenarios, exploring plausible land management outcomes in the future. The authors believed that residents were well-positioned to evaluate the impacts of changing land management on their lives. Yet, scenario-based interviews have several limitations. First, a respondent’s ability to accurately predict their actions may decline as the scenarios become more complex^44^. However, relatively simple scenarios were presented, which were extensions of current land management practices. Second, these evaluations may not have fully accounted for indirect, unintuitive, or hidden feedback that might affect residents in complex socio-ecological systems. For instance, preventing hunting might lead to increased populations of crop-raiding wildlife, further harming food security. Finally, respondents may provide strategic responses if they believe this will influence the choice of intervention. Therefore, respondents were informed that no interventions were planned as part of this research. In general, a large body of research explores the limitations of expert elicitation methods in conservation and related fields (e.g.^78^). Many of these limitations might also apply when using scenario-based interviews, suggesting the potential value of structured elicitation protocols when using these interviews (e.g.^79^). Equally, a researcher’s positionality may influence which and how scenarios are presented, how participants respond, and how the results are interpreted. Therefore, reflexive research practices may be needed when using these methods, as in other conservation and sustainability studies^80,81^. Finally, while there is increasing application of scenario-based interviews in conservation and related fields (e.g.^44,45^), there appears to be limited evidence testing how well respondents’ predictions perform against actual interventions.

It was assumed that psychobiological vulnerability varied randomly with respect to the exposure variables, such as economic poverty. However, there can be dynamic bi-directional feedback between mental illness and life stressors over an individual’s life course. For instance, previous research in the study area suggested that “thinking too much” was linked with impaired livelihood activities, potentially worsening social determinants of psychological distress^14^. These dynamic feedbacks represent an opportunity for future time-series and cohort research, exploring how landscapes, livelihoods, and mental health co-evolve over time.

The study population was restricted to farming households and did not consider the impacts of land management on the mental health of other groups. However, contract farming can have complex effects on non-farmers, such as labourers^42,43^. The expansion of sugarcane farming in the study area has provided jobs for landless residents and migrants^50^, so might have supported the mental health of these groups. These potential benefits should be understood and accounted for when weighing the suitability of different land management approaches.

The PHQ-8 has been used internationally and is based on an instrument that has been used in Uganda^54,55^. However, western diagnostic criteria and psychological concepts might not be universally appropriate and overlook culturally relative syndromes^58^. Prior research in the study area suggested partial overlap in symptoms associated with “thinking too much” and depression^14^. The current study found a strong association between depressive symptom severity, estimated using the PHQ-8, and reported experiences of “thinking too much”. Moreover, “thinking too much” had similar associations with the explanatory variables as found in the primary analysis. This result suggests that the findings and their broader policy implications are unlikely to be an artefact of using a western diagnostic tool.

### Policy implications for land management and mental health

Common mental disorders are a leading cause of morbidity and disability^11^, but there has been a “collective failure to respond to this global health crisis”^10^. Recognising this, the field of global mental health has emerged, which seeks to enhance both the treatment and prevention of mental illness worldwide^82^. As part of this prevention, there have been recent calls to better understand and manage the social, cultural, and economic causes of mental illness. For instance, Collins et al.^83^ highlights the need to better “support community environments that promote physical and mental well-being throughout life”. However, the results here suggest the need to look beyond the social context to understand distal socio-ecological factors that may indirectly influence mental health. These factors may include how landscape management can influence social determinants of mental illness. Moreover, this study responds to calls for forward-looking predictive landscape planning^84^. In doing so, the study suggests potential trade-offs that should be accounted for within land management for holistic sustainable development.

Protected areas will remain a core part of global conservation efforts, with the “30 by 30” plan to nearly double their current extent over the next decade^4^. The social impacts of this expansion may vary depending on residents’ context-dependent relationships with nature and access rules. However, the results of this study suggest restrictive protected areas may threaten the mental health of those whose livelihoods depend on access to nature. In these contexts, gazetting strictly protected areas might undermine progress towards health (SDG 3) and other sustainable development goals among some groups. However, protected areas are unlikely to be unambiguously bad for mental health. For example, Buckley et al.^85^ estimated that the economic value of improved mental health among visitors to protected areas was several orders of magnitude greater than their management budgets. Nevertheless, many residents of biodiverse countries depend on nature for their basic needs while being under-served by mental healthcare services^86,87^. So, promoting the expansion of protected areas in these places might exacerbate social determinates of mental illness among already vulnerable populations. Recognising this, governments might explore alternatives to strictly protected areas. While this study did not evaluate these alternatives, the results suggest the potential benefits of fostering sustainable forest use. In Uganda, this could include strengthening and promoting collaborative forest management agreements, a legal mechanism with dual aims of enhancing livelihoods while conserving forests^88^. More broadly, mounting evidence shows that Indigenous groups and local communities play vital roles in protecting nature^89,90^. So governments, conservation organisations, and other actors might consider ways to support residents to use nature sustainably. This support could include, for example, strengthening residents’ collective land tenure, self-determined governance systems, and their ability to defend against external drivers of nature loss^38^. Further research is needed to evaluate how land management approaches can effectively support residents’ mental health. However, such alternatives might help prevent mental illness (SDG 3) while meeting other conservation and sustainable development goals.

Contract farming has been promoted as a tool for more inclusive agricultural development, including within Uganda^40,91^. In general, the extent to which contract farming contributes to equitable sustainable development depends on how well it engages small-scale farmers^40^. Further research is needed to understand if well-designed pro-poor contract farming policies can support residents’ mental health. However, these policies could include making contract farming more inclusive, such as prioritising agricultural extension services to the poorest in communities. Yet, more inclusive contract farming may not always be feasible, suggesting the value of alternative options for the poorest in communities. In the current study area, this might include promoting collaborative forest management with households with limited land. Moreover, land managers might consider ways to reduce adverse spillover effects, such as enhancing tenure by making it easier and cheaper for poorer households to claim land titles.

## Conclusion

This study shows how the expansion of strictly protected areas may harm the mental health of forest users. Furthermore, the study illustrates how contract-farming approaches that worsen inequitable land distributions may harm the mental health of small-scale farmers. Therefore, decision-makers seeking to support mental health should be wary of approaches that impair livelihood activities by restricting access to nature. However, health is only one of the multiple sustainable development objectives, and the links between landscapes and wellbeing can be complex and context-dependent. Given this complexity, navigating trade-offs and synergies offered by different land management approaches can be challenging. Future research is needed to explore how landscape management could support mental health alongside other societal priorities. For example, future research could evaluate whether measures promoting locally-led conservation or sustainable and inclusive agricultural intensification can support residents’ mental health and whether such measures are cost-effective compared to existing public mental health interventions. It may also be useful to examine the accuracy of predictions from scenario-based interviews using long-term social and ecological studies. Future research might also explore potential bi-directional feedback between people’s mental health and interactions with nature using cohort studies, which are rare in conservation and sustainability research.

Several principles might help in this process. First, a “whole-of-government” approach (encouraging dialogue and strategic planning across government agencies^4^) may help promote coherent and coordinated sustainable development policy. For example, this could include exploring if sustainable forest use interventions could be a tool for preventing mental illness. Second, decision-makers, practitioners, and researchers should be wary of one-size-fits-all solutions, such as plans focused on expanding protected areas. Instead, a “whole-of-society” approach (strengthening the inclusion and participation of diverse societal actors^4^) might help find locally appropriate and socially just landscape solutions. This approach could include scenario-based stakeholder engagement for inclusive, transparent, and forward-looking landscape planning. These and other approaches may help reduce the immense global burden of mental illness, supporting sustainable development and living in harmony with nature.

## Supplementary Information

Supplementary Information.

## Data Availability

The anonymised data used in the statistical analysis is available from Figshare (https://doi.org/10.6084/m9.figshare.16955221). The code used in the statistical analysis is available from: https://github.com/Pienkowski/Uganda_quant_analysis_pub. Please contact the authors directly for the scenario data.
