# Supplementary material for: Predicting the impacts of land management for sustainable development on depression risk in a Ugandan case study
Source: Sci Rep. 2022 Jul 8;12:11607. doi: 10.1038/s41598-022-14976-3 (PMC9270416; doi:10.1038/s41598-022-14976-3)
Supplement: Supplementary file 1 — Supplementary Information. [file 41598_2022_14976_MOESM1_ESM.docx]

# Predicting the impacts of land management for sustainable development on depression risk in a Ugandan case study

Thomas Pienkowski^a,1,^, Aidan Keane^b^, Eugene Kinyanda^c^, Caroline Asiimwe^d,e^, and E.J. Milner-Gulland^a^

^a^ Department of Zoology, University of Oxford, Oxford, OX1 3SZ, UK

^b^ School of GeoSciences, University of Edinburgh, Edinburgh, EH9 3FF, UK

^c^ Medical Research Council/Uganda Virus Research Institute and London School of Hygiene & Tropical Medicine Uganda Research Unit, 51-59 Nakiwogo Street, Entebbe, Uganda

^d^ Budongo Conservation Field Station, Masindi, Uganda

^e^ Jane Goodall Institute, Plot 26 Lugard Avenue, P.O. Box 462, Entebbe, Uganda

^1^ thomas.pienkowski@zoo.ox.ac.uk

## Supplementary methods 1: Survey information and questions

The survey information as read to participants prior to starting the survey and seeking their consent. The survey was delivered through electronic tablets using the ODK software. Conditional logic was used to ask paired questions. For example, the first part of question 1.9.a asks “How is your health in general? Is your health good, fair, or bad?”. If the respondent says “good”, then the participant is then asked “Is your health very good or just good?” (question 1.9.a). Similar conditional logic was used for many of the Likert-scaled items and in the sets of scenario questions. This approached was used to reduce the cognitive load of each question and to ensure consistent in delivery.

**Survey information**

**Introduction**

This study is being conducted by the Interdisciplinary Centre for Conservation Science (ICCS) at the University of Oxford in collaboration with MRC/UVRI & LSHTM Uganda Research Unit.

This document is called the participant information sheet and contains information to help you decide if you would like to participate in the study. Joining the study is entirely up to you. The study staff will talk with you about this information and answer any questions that you may have. Please feel free to talk to others about this study if you wish.

**Before we start**

Before we start, I would like to read out some symptoms that are related to COVID-19. I would like you to tell me if you or any of your family have had any of these symptoms in the past week:

a high temperature – this means you feel hot to touch on your chest or back (you do not need to measure your temperature)

a new, continuous cough – this means coughing a lot for more than an hour, or 3 or more coughing episodes in 24 hours (if you usually have a cough, it may be worse than usual)

a loss or change to your sense of smell or taste – this means you've noticed you cannot smell or taste anything, or things smell or taste different to normal

Have you had any of these symptoms? [*If yes, follow the ‘Closing an interview or survey’ procedure. If no, proceed with the survey information form.*]

**What is the Purpose of the study?**

The ICCS and MRC/UVRI & LSHTM Uganda Research Unit is conducting research to understand the things that people find difficult in their lives, in these communities. We are most interested in things to do with the environment around you.

**Why have I been asked to take part?**

You have been asked to take part because you are the male or female head of the household in a community we are collecting data in.

**Do I have to take part?**

You do not have to take part in the research or answer any of the questions and are free to stop anytime.

**What will happen to me if I take part?**

We want to ask you a set of questions about yourself and the challenges you face.

**What will I have to do?**

If you decide to take part, then we will sit together, and I will ask you questions, which can choose to answer or not answer.

**What are the possible risks and inconveniences?**

Since we will be asking about some of the challenges people in your community face, you may find the conversation upsetting or distressing. The chat is expected to take around 30 minutes.

**What are the possible benefits?**

There are no direct benefits from taking part, and nothing is expected to change in the community because of the research. You will not be paid to take part, but you will be compensated for your time (discussed below).

**How many participants will be enrolled in this study?**

We are speaking to 708 people in the area.

**For how long will I have to participate in the study?**

Participation lasts for as long as the interview takes.

**Can I change my mind about participating in the study?**

You can withdraw from the study at any time. You can stop and withdraw from the study by telling us at any time during the survey. You can also withdraw later on by contacting us on here *[provide contact sheet].*

**What will happen to information collected about me?**

All information collected about you will be kept private. Only the study staff and authorities who check that the study is being carried out properly will be allowed to look at information about you. Data may be sent to other study staff at the University of Edinburgh, but this will be anonymized. This means that any information about you which leaves the study site will have your name and address removed so that you cannot be recognized.

Your personal details will be kept in a different safe place to the other study information and will be destroyed at the end of the study. The data will be made available to other researchers worldwide for research and to improve medical knowledge and patient care. Your personal information will not be included, and there is no way that you can be identified.

**What happens to the results of the study?**

We will be conducting a meeting with community leaders to tell them about the results of the study. The study results will also be published in a journal so that other researchers can learn from them. Your personal information will not be included in the study report, and there is no way that you can be identified from it.

**What other choices do I have besides participating in this study?**

You can freely choose not to take part in the study. Choosing not to participate will not affect you in any way.

**Will it cost you anything to take part in this study?**

There is no cost to you for participating.

**Will you be paid to take part in this study?**

No, you will not be paid for participating in this study. However, you will be given compensation (including oil, tea, soap, sugar and salt worth UGX 15,000) for your time.

**What will happen if I get injured as a result of participation in this study?**

We do not anticipate any injury as a result of your participation in this research study. However, if you become ill or injured during your participation in this study, please inform the study staff immediately. The study staff will inform you where you can get additional treatment when required.

**Who has allowed this research to take place?**

This study was first reviewed by the Unit’s Scientific Committee prior to submission to an independent Research ethics committee (UVRI-REC) and Uganda National Council for Science and Technology (UNCST) who have looked carefully at this work and agreed that the research is important, it will be conducted properly and participants’ safety and rights have been respected. The study was also approved by the University of Oxford Central University Research Ethics Committee (reference number: R63458/RE001).

**Whom should you call if you have questions regarding this study or if you get an injury?**

If you have any questions about this study or a study-related injury, please contact Thomas Pienkowski, the principle investigator of this study on telephone number [redacted]*/*WhatsApp: [redacted] or Professor Eugene Kinyanda a lead supervisor of this study on telephone number [redacted].

If you have any questions about your rights as a research participant, please contact: Mr. Tom Lutalo chairman of the UVRI-REC on telephone numbers [redacted].

**Section 1: Demographics & general health**

|  | **QUESTION** | **RESPONSE** |
| --- | --- | --- |
| 0 | Interviewer ID  [NOTE] Your initials | MM  GG  LA  SL  JR |
| 1.1 | Study I.D. Number | …………………… |
| 1.2 | Location (*in-built GPS*)  [NOTE] Wait for "Using GPS. Accuracy is…" to appear, then click "Save GeoPoint". | …………………… |
| 1.3 | Community | Nyabyeya T.C.  Nyabyeya One  Nyabyeya Two  Nyabigoma  Nyakafunjo  Kyempunu  Korongo  Maramu  Ewafala  Kadukuru One  Kadukuru Two |
| 1.4 | Date of interview | dd / mm / yy  ……/……/…… |
| 1.5 | Sex? | Male  Female  Refused/other |
| 1.6 | How many years old are you?  [NOTE] The respondent must between 18 and 60. | ……………………yrs  Don’t Know  Refused |
| 1.7 | Where did you stop in your education? | No education  Started primary  Finished primary  Started secondary  Finished secondary  Beyond secondary  Don’t Know  Refused |
| 1.8 | What is your marital status? Are you… | Single  Married once  Polygamist  Divorced or separated  I lost my husband or wife  Don’t Know  Refused |
| 1.9.a | How is your health in general? Is your health good, fair, or bad? | Good  Fair  Bad  Don’t Know  Refused |
| 1.9.b | [If good] Is your health very good or just good? | Very good  Good  Don’t Know  Refused |
| 1.9.c | [If bad] Is your health very bad or just bad? | Very bad  Bad  Don’t Know  Refused |
| 1.10 | How many children under 18 live in your household?  [NOTE] Under 18, living here. | …………………… |
| 1.11 | How many adults 18 or over live in your household?  [NOTE] 18 and above, living here. Including yourself. | …………………… |
| 1.12 | What is the main source of money in your household? | Farming  Cattle keeping  Trapping fish  Worker (like cutting sugar cane or weeding, driver)  Own business  Other  Don’t Know  Refused |

**Section 2: Socioeconomic status, farming, and forest**

|  | **QUESTION** | | | **RESPONSE** | |
| --- | --- | --- | --- | --- | --- |
| 2.1 | I am now going to read to you some things. For each thing I am going to read to you, I would like you to tell me if your household has it.  Does your household have…? | | | | |
|  |  | | Yes | | No |
| 2.1.1 | A gas cooker | |  | |  |
| 2.1.2 | At least two sets of clothes for every person in the household | |  | |  |
| 2.1.3 | Two goats | |  | |  |
| 2.1.4 | At least two meals per day | |  | |  |
| 2.1.5 | Mains electricity | |  | |  |
| 2.1.6 | A wooden bed (frame) | |  | |  |
| 2.1.7 | A big solar panel | |  | |  |
| 2.1.8 | At least two Jerry cans | |  | |  |
| 2.1.9 | One mattress or more | |  | |  |
| 2.1.10 | A television | |  | |  |
| 2.1.11 | Brick walls for your house | |  | |  |
| 2.1.12 | A motorbike | |  | |  |
| 2.1.13 | Two hoes | |  | |  |
| 2.1.14 | A blanket for every person in the household | |  | |  |
| 2.1.15 | A bank account | |  | |  |
| 2.1.16 | A bicycle | |  | |  |
| 2.1.17 | A water tank | |  | |  |
| 2.1.18 | A pair of shoes for every member of the household | |  | |  |
| 2.1.19 | Iron sheets on your roof | |  | |  |
| 2.1.20 | A radio | |  | |  |
| 2.1.21 | Three saucepans | |  | |  |
| 2.1.22 | Solar battery | |  | |  |
| 2.1.23 | A mobile phone | |  | |  |
| 2.1.24 | Two or more plastic chairs | |  | |  |
| 2.1.25 | A car | |  | |  |
| 2.1.26 | A fridge | |  | |  |
| 2.1.27 | A cement floor for your house | |  | |  |
| 2.1.28 | A member in a savings group | |  | |  |
| 2.1.29 | A sofa | |  | |  |
| 2.1.30 | Two or more wooden chairs | |  | |  |
| 2.1.31 | Soap | |  | |  |
| 2.2.a | In the past month, how hard has it been for you to pay for the things you need the most like food, medicine, or clothing? Was it hard or not hard? | Hard  Not hard  Don’t Know  Refused | | | |
| 2.2.b | [If hard] Was it very hard, hard, or a bit hard? | Very hard  Hard  A bit hard  Don’t Know  Refused | | | |
| 2.3 | I would now like to ask you about your farm. | | | | |
| 2.3.1.a | Thinking about your farmland, how much land does your household have? Count all the land you own and rent together. Is it a small amount of land, a large amount of land, or in the middle? | Small land  In the middle  Large land  Don’t Know  Refused | | | |
| 2.3.1.b | [If small] It is a very small amount of land or just a small amount of land? | Very small land  Small land  Don’t Know  Refused | | | |
|  | [If large] It is a very large amount of land or just a large amount of land? | Very large land  Large land  Don’t Know  Refused | | | |
| 2.3.2a | Can you tell me in plots, or acres or hectares how much land your household has for farming? Count any owned or rented land together.  [NOTE] Put 98 if does not know. One and a half is 1.5, a half is 0.5, a third is 0.33, and quarter is 0.25. | …………………… | | | |
| 2.3.2b | What unit?  [NOTE] Did they say it in plots, acres, or hectares? Put 98 if does not know. (4 plots = 1 acre & 2 and ½ acre = 1 hectare). | Plot  Acre  Hectare  Don’t Know  Refused | | | |
| 2.4 | Does your household grow sugarcane for selling?  [NOTE] Selling to the factory. | Yes  No  Don’t Know  Refused | | | |
| 2.5 | We would like to hear how you feel about some sayings. These sayings are going to be read to you. We would like you to tell us if you agree or disagree with these sayings using the answers given. Have you understood? | | | | |
| 2.5.1.a | Your household's farm is bigger than most others in this community.  Do you agree, you disagree, or you are in the middle? | Agree  In the middle  Disagree  Don’t Know  Refused | | | |
| 2.5.1.b | [If agree] Do you agree a lot, or you agree a little? | Agree a lot  Agree a little  Don’t Know  Refused | | | |
| 2.5.1.b | [If disagree] Do you disagree a lot, or you disagree a little? | Disagree a lot  Disagree a little  Don’t Know  Refused | | | |
| 2.5.2.a | Your household's farm is big enough for you to get the things you need, like food, school fees, or medicine. Do you agree, you disagree, or you are in the middle?  Do you agree, you disagree, or you are in the middle? | Agree  In the middle  Disagree  Don’t Know  Refused | | | |
| 2.5.2.b | Do you agree a lot, or you agree a little? | Agree a lot  Agree a little  Don’t Know  Refused | | | |
| 2.5.2.c | Do you disagree a lot, or you disagree a little? | Disagree a lot  Disagree a little  Don’t Know  Refused | | | |
| 2.5.3.a | Your household struggles because you do not have enough land.  Do you agree, you disagree, or you are in the middle? | Agree  In the middle  Disagree  Don’t Know  Refused | | | |
| 2.5.3.b | Do you agree a lot, or you agree a little? | Agree a lot  Agree a little  Don’t Know  Refused | | | |
| 2.5.3.c | Do you disagree a lot, or you disagree a little? | Disagree a lot  Disagree a little  Don’t Know  Refused | | | |
| 2.5.4.a | Your household has enough land to live well.  Do you agree, you disagree, or you are in the middle? | Agree  In the middle  Disagree  Don’t Know  Refused | | | |
| 2.5.4.b | Do you agree a lot, or you agree a little? | Agree a lot  Agree a little  Don’t Know  Refused | | | |
| 2.5.4.c | Do you disagree a lot, or you disagree a little? | Disagree a lot  Disagree a little  Don’t Know  Refused | | | |
| 2.5.5.a | Your household gets good money from your farm. | Agree  In the middle  Disagree  Don’t Know  Refused | | | |
| 2.5.5.b | Do you agree a lot, or you agree a little? | Agree a lot  Agree a little  Don’t Know  Refused | | | |
| 2.5.5.c | Do you disagree a lot, or you disagree a little? | Disagree a lot  Disagree a little  Don’t Know  Refused | | | |
| 2.5.6.a | Your household's farm is smaller than most others in this community.  Do you agree, you disagree, or you are in the middle? | Agree  In the middle  Disagree  Don’t Know  Refused | | | |
| 2.5.6.b | Do you agree a lot, or you agree a little? | Agree a lot  Agree a little  Don’t Know  Refused | | | |
| 2.5.6.c | Do you disagree a lot, or you disagree a little? | Disagree a lot  Disagree a little  Don’t Know  Refused | | | |
| 2.6 | I would now like to ask you about the things you get from the forest. Like before, we would like to hear how you feel about some sayings. | | | | |
| 2.6.1.a | Your household gets things from the forest that help you a lot.  Do you agree, you disagree, or you are in the middle? | Agree  In the middle  Disagree  Don’t Know  Refused | | | |
| 2.6.1.b | Do you agree a lot, or you agree a little? | Agree a lot  Agree a little  Don’t Know  Refused | | | |
| 2.6.1.c | Do you disagree a lot, or you disagree a little? | Disagree a lot  Disagree a little  Don’t Know  Refused | | | |
| 2.6.2 | Your household gets good money from things in the forest.  Do you agree, you disagree, or you are in the middle? | Agree  In the middle  Disagree  Don’t Know  Refused | | | |
| 2.6.2.a | Do you agree a lot, or you agree a little? | Agree a lot  Agree a little  Don’t Know  Refused | | | |
| 2.6.2.b | Do you disagree a lot, or you disagree a little? | Disagree a lot  Disagree a little  Don’t Know  Refused | | | |
| 2.6.3.a | The forest helps you buy things you need, like food, school fees, or medicine.  Do you agree, you disagree, or you are in the middle? | Agree  In the middle  Disagree  Don’t Know  Refused | | | |
| 2.6.3.b | Do you agree a lot, or you agree a little? | Agree a lot  Agree a little  Don’t Know  Refused | | | |
| 2.6.3.c | Do you disagree a lot, or you disagree a little? | Disagree a lot  Disagree a little  Don’t Know  Refused | | | |
| 2.6.4.a | It would be very bad for your household if you could not get things from the forest.  Do you agree, you disagree, or you are in the middle? | Agree  In the middle  Disagree  Don’t Know  Refused | | | |
| 2.6.4.b | Do you agree a lot, or you agree a little? | Agree a lot  Agree a little  Don’t Know  Refused | | | |
| 2.6.4.c | Do you disagree a lot, or you disagree a little? | Disagree a lot  Disagree a little  Don’t Know  Refused | | | |
| 2.6.5.a | Your household gets food from the forest.  Do you agree, you disagree, or you are in the middle? | Agree  In the middle  Disagree  Don’t Know  Refused | | | |
| 2.6.5.b | Do you agree a lot, or you agree a little? | Agree a lot  Agree a little  Don’t Know  Refused | | | |
| 2.6.5.c | Do you disagree a lot, or you disagree a little? | Disagree a lot  Disagree a little  Don’t Know  Refused | | | |
| 2.6.6.a | Getting things from the forest helps your household to survive.  Do you agree, you disagree, or you are in the middle? | Agree  In the middle  Disagree  Don’t Know  Refused | | | |
| 2.6.6.b | Do you agree a lot, or you agree a little? | Agree a lot  Agree a little  Don’t Know  Refused | | | |
| 2.6.6.c | Do you disagree a lot, or you disagree a little? | Disagree a lot  Disagree a little  Don’t Know  Refused | | | |
| 2.6.7.a | Your household would have no money or food if you could not go to the forest.  Do you agree, you disagree, or you are in the middle? | Agree  In the middle  Disagree  Don’t Know  Refused | | | |
| 2.6.7.b | Do you agree a lot, or you agree a little? | Agree a lot  Agree a little  Don’t Know  Refused | | | |
| 2.6.7.c | Do you disagree a lot, or you disagree a little? | Disagree a lot  Disagree a little  Don’t Know  Refused | | | |
| 2.7 | Is any part of your farm beside the forest? | Yes  No  Don’t Know  Refused | | | |

**Section 3: Food Insecurity Experience Scale (FIES)**

|  | **QUESTION** | | **RESPONSE** |
| --- | --- | --- | --- |
| 3 | Now I would like to ask you some questions about food. | | |
| 3.1 | During the last 3 months, was there a time when you or others in your household worried about not having enough food to eat because of a lack of money or other resources?  [NOTE] Last 3 months | Yes  No  Don’t Know  Refused | |
| 3.2 | Still thinking about the last 3 months, was there a time when you or others in your household were unable to eat healthy and nutritious food because of a lack of money or other resources?  [NOTE] Last 3 months | Yes  No  Don’t Know  Refused | |
| 3.3 | Was there a time when you or others in your household ate only a few kinds of foods because of a lack of money or other resources?  [NOTE] Last 3 months | Yes  No  Don’t Know  Refused | |
| 3.4 | Was there a time when you or others in your household had to skip a meal because there was not enough money or other resources to get food?  [NOTE] Last 3 months | Yes  No  Don’t Know  Refused | |
| 3.5 | Still thinking about the last 3 months, was there a time when you or others in your household ate less than you thought you should because of a lack of money or other resources?  [NOTE] Last 3 months | Yes  No  Don’t Know  Refused | |
| 3.6 | Was there a time when your household ran out of food because of a lack of money or other resources?  [NOTE] Last 3 months | Yes  No  Don’t Know  Refused | |
| 3.7 | Was there a time when you or others in your household were hungry but did not eat because there was not enough money or other resources for food?  [NOTE] Last 3 months | Yes  No  Don’t Know  Refused | |
| 3.8 | Was there a time when you or others in your household went without eating for a whole day because of a lack of money or other resources?  [NOTE] Last 3 months | Yes  No  Don’t Know  Refused | |

**Section 4: Multidimensional Scale of Perceived Social Support (MSPSS) – 6-item**

|  | **QUESTION** | | **RESPONSE** |
| --- | --- | --- | --- |
| 4 | We would like to hear how you feel about some sayings. These sayings are going to be read to you. We would like you to tell us if you agree or disagree with these sayings using the answers given. Have you understood? | | |
| 4.1.a | There is a special person around you when you are in need.  Do you agree, you disagree, or you are in the middle? | Agree  In the middle  Disagree  Don’t Know  Refused | |
| 4.1.b | Do you disagree a lot, or you disagree a little? | Agree a lot  Agree a little  Don’t Know  Refused | |
| 4.1.c | Is any part of your farm beside the forest? | Disagree a lot  Disagree a little  Don’t Know  Refused | |
| 4.2.a | Your family tries to help you.  Do you agree, you disagree, or you are in the middle? | Agree  In the middle  Disagree  Don’t Know  Refused | |
| 4.2.b | Do you disagree a lot, or you disagree a little? | Agree a lot  Agree a little  Don’t Know  Refused | |
| 4.2.c | Is any part of your farm beside the forest? | Disagree a lot  Disagree a little  Don’t Know  Refused | |
| 4.3.a | You get the comfort and support you need from your family.  Do you agree, you disagree, or you are in the middle? | Agree  In the middle  Disagree  Don’t Know  Refused | |
| 4.3.b | Do you disagree a lot, or you disagree a little? | Agree a lot  Agree a little  Don’t Know  Refused | |
| 4.3.c | Is any part of your farm beside the forest? | Disagree a lot  Disagree a little  Don’t Know  Refused | |
| 4.4.a | You know your friends will be around to help when trouble finds you.  Do you agree, you disagree, or you are in the middle? | Agree  In the middle  Disagree  Don’t Know  Refused | |
| 4.4.b | Do you disagree a lot, or you disagree a little? | Agree a lot  Agree a little  Don’t Know  Refused | |
| 4.4.c | Is any part of your farm beside the forest? | Disagree a lot  Disagree a little  Don’t Know  Refused | |
| 4.5.a | You can talk about your problems with your friends.  Do you agree, you disagree, or you are in the middle? | Agree  In the middle  Disagree  Don’t Know  Refused | |
| 4.5.b | Do you disagree a lot, or you disagree a little? | Agree a lot  Agree a little  Don’t Know  Refused | |
| 4.5.c | Is any part of your farm beside the forest? | Disagree a lot  Disagree a little  Don’t Know  Refused | |
| 4.6.a | There is a special person with whom you can share joys and sorrows.  Do you agree, you disagree, or you are in the middle? | Agree  In the middle  Disagree  Don’t Know  Refused | |
| 4.6.b | Do you disagree a lot, or you disagree a little? | Agree a lot  Agree a little  Don’t Know  Refused | |
| 4.6.c | Is any part of your farm beside the forest? | Disagree a lot  Disagree a little  Don’t Know  Refused | |

**Section 5: Alcohol use and smoking**

|  | **QUESTION** | **RESPONSE** |
| --- | --- | --- |
| 5.1.1 | Do you take alcohol? | Yes  No  Don’t Know  Refused |
| 5.1.2 | How many days a week?  [NOTE] 0 = less than once a week, 98 = don't know, 99 = refuse | ……………………times a week |
| 5.2 | Do you smoke tobacco every day? | Yes  No  Don’t Know  Refused |

**Section 6: Patient Health Questionnaire (PHQ-8)**

|  | **QUESTION** | | **RESPONSE** |
| --- | --- | --- | --- |
| 6 | I would now like to ask you some questions about how you feel. | | |
| 6.1 | Over the past two weeks, how many times were you disturbed by little interest or pleasure in doing things? Was it not at all, a few days, more than half the days, or nearly every day? | 0 = Not at all  1 = Few days  2 = More than half the days  3 = Nearly every day  NA = Don’t Know | |
| 6.2 | Over the past two weeks, how many times were you disturbed by feeling down, unhappy, or like you cannot see a way forward? Was it not at all, a few days, more than half the days, or nearly every day? | 0 = Not at all  1 = Few days  2 = More than half the days  3 = Nearly every day  NA = Don’t Know | |
| 6.3 | Over the past two weeks, how many times were you disturbed by trouble falling or staying asleep, or sleeping too much? Was it not at all, a few days, more than half the days, or nearly every day? | 0 = Not at all  1 = Few days  2 = More than half the days  3 = Nearly every day  NA = Don’t Know | |
| 6.4 | Over the past two weeks, how many times were you disturbed by feeling tired or having little energy? Was it not at all, a few days, more than half the days, or nearly every day? | 0 = Not at all  1 = Few days  2 = More than half the days  3 = Nearly every day  NA = Don’t Know | |
| 6.5 | Over the past two weeks, how many times were you disturbed by not wanting to eat or eating too much? Was it not at all, a few days, more than half the days, or nearly every day? | 0 = Not at all  1 = Few days  2 = More than half the days  3 = Nearly every day  NA = Don’t Know | |
| 6.6 | Over the past two weeks, how many times were you disturbed by feeling bad about yourself, or you are a failure, or you have failed yourself or your family? Was it not at all, a few days, more than half the days, or nearly every day? | 0 = Not at all  1 = Few days  2 = More than half the days  3 = Nearly every day  NA = Don’t Know | |
| 6.7 | Over the past two weeks, how many times were you disturbed by challenges putting your mind on what you are doing, like a church service or speaking with people? Was it not at all, a few days, more than half the days, or nearly every day? | 0 = Not at all  1 = Few days  2 = More than half the days  3 = Nearly every day  NA = Don’t Know | |
| 6.8 | Over the past two weeks, how many times were you disturbed by moving or speaking so slowly that other people notice? Or in the opposite way, fidgeting so much that you move a lot more than how you used to do? Was it not at all, a few days, more than half the days, or nearly every day? | 0 = Not at all  1 = Few days  2 = More than half the days  3 = Nearly every day  NA = Don’t Know | |
|  | TOTAL score (sum items 6.1 to 6.8) | ……………………………… | |
| 6.9 | Over the past two weeks, how many times were you disturbed by thinking to much or too many thoughts? Was it not at all, a few days, more than half the days, or nearly every day? | 0 = Not at all  1 = Few days  2 = More than half the days  3 = Nearly every day  NA = Don’t Know | |

**Section 7a: Scenario set 1**

|  | **QUESTION** | **RESPONSE** |
| --- | --- | --- |
|  | We have nearly finished our questions. When we ask you these questions, we would like to think about the next ten years. You might not know the answer, but I would like you to think about what could happen. We want to remind you that there is no plan or project coming from this research. Do you understand? | |
| 7a.1.1.a | Some households in this community get food from the forest, like herbs, animals and mushrooms. In the next ten years, do you think households will get less food from the forest, more food from the forest, or will there be no change? | Less food  No change  More food  Don’t Know  Refused |
| 7a.1.1.b | [If less] Why is this?  [NOTE] Let the respondent answer first. If they don't know, then read out the responses. Select all that apply. | Because the guards will stop them  Because of over-hunting  Because there is less forest/less food for animals  Because of disease  Other reasons  Don’t Know  Refused |
| 7a.1.1.c | [If more] Why is this?  [NOTE] Let the respondent answer first. If they don't know, then read out the responses. Select all that apply. | Because there are more people  Because of more hunger  Because of more poverty  Because people want to eat more of those foods  Because there will be more food in the forest  Other reasons  Don’t Know  Refused |
| 7a.1.1.d | [If other from SEVAFWHYA or SEVAFWHYB] Write a few words summarising the response, in English. | …………………… |
| 7a.1.2 | If households get less food from the forest in the next ten years, would there be more hunger, less hunger, or no change? | More hunger  No change  Less hunger  Don’t Know  Refused |
| 7a.1.3 | If households get more food from the forest in the next ten years, would there be more hunger, less hunger, or no change? | More hunger  No change  Less hunger  Don’t Know  Refused |
| 7a.2.1.a | Some households in this community get things from the forest to make money, like selling firewood, timber, and charcoal. In the next ten years, do you think households will get fewer things from the forest to make money, more things from the forest to make money, or will there be no change? | More things  No change  Fewer things  Don’t Know  Refused |
| 7a.2.1.b | [If less] Why is this?  [NOTE] Let the respondent answer first. If they don't know, then read out the responses. Select all that apply. | Because the guards will stop them  Because of over harvesting  Because the forest will become farmland  Because of disease  Other reasons  Don’t Know  Refused |
| 7a.2.1.c | [If more] Why is this?  [NOTE] Let the respondent answer first. If they don't know, then read out the responses. Select all that apply. | Because there are more people  Because of more hunger  Because of more poverty  Because people want to get more money  Because there will be more things in the forest for money  Other reasons  Don’t Know  Refused |
| 7a.2.1.d | [If other from SEVAMONWHYA  or SEVAMONWHYB] Write a few words summarising the response, in English. | …………………… |
| 7a.2.2 | If households get fewer things for money from the forest in the next ten years, would there be more poverty, less poverty, or no change? | More poverty  No change  Less poverty  Don’t Know  Refused  Less food |
| 7a.2.3 | If households get more things for money from the forest in the next ten years, would there be more poverty, less poverty, or no change? | More poverty  No change  Less poverty  Don’t Know  Refused  Less food |

**Section 7b: Scenario set 2**

|  | **QUESTION** | **RESPONSE** |
| --- | --- | --- |
|  | We have nearly finished our questions. When we ask you these questions, we would like to think about the next ten years. You might not know the answer, but I would like you to think about what could happen. We want to remind you that there is no plan or project coming from this research. Do you understand? | |
| 7b.1.1.a | Some households in this community get food from the forest, like herbs, animals and mushrooms. I want you to imagine that people were not allowed to get anything from the forest, and there were more guards in the forest, over the next ten years. When you imagine this, do you think households would get less food from the forest, more food from the forest, or would there be no change? | Less food  No change  More food  Don’t Know  Refused |
| 7b.1.1.b | [If less] Why is this?  [NOTE] Let the respondent answer first. If they don't know, then read out the responses. Select all that apply. | Because the guards will stop them  Because of over-hunting  Because there is less forest/less food for animals  Because of disease  Other reasons  Don’t Know  Refused |
| 7b.1.1.c | [If more] Why is this?  [NOTE] Let the respondent answer first. If they don't know, then read out the responses. Select all that apply. | Because there are more people  Because of more hunger  Because of more poverty  Because people want to eat more of those foods  Because there will be more food in the forest  Other reasons  Don’t Know  Refused |
| 7b.1.1.d | [If other from SEVBFWHYA or SEVBFWHYB] Write a few words summarising the response, in English. | …………………… |
| 7b.1.2 | If households get less food from the forest in the next ten years, would there be more hunger, less hunger, or no change? | More hunger  No change  Less hunger  Don’t Know  Refused |
| 7b.1.3 | If households get more food from the forest in the next ten years, would there be more hunger, less hunger, or no change? | More hunger  No change  Less hunger  Don’t Know  Refused |
| 7b.2.1 | Some households in this community get things from the forest to make money, like selling firewood, timber, and charcoal. I want you to still imagine that people were not allowed to get anything from the forest, and there were more guards in the forest, over the next ten years. When you imagine this, do you think people would get fewer things from the forest to make money, more things from the forest to make money, or would there be no change? | More things  No change  Fewer things  Don’t Know  Refused |
|  | [If less] Why is this?  [NOTE] Let the respondent answer first. If they don't know, then read out the responses. Select all that apply. | Because the guards will stop them  Because of over harvesting  Because the forest will become farmland  Because of disease  Other reasons  Don’t Know  Refused |
|  | [If more] Why is this?  [NOTE] Let the respondent answer first. If they don't know, then read out the responses. Select all that apply. | Because there are more people  Because of more hunger  Because of more poverty  Because people want to get more money  Because there will be more things in the forest for money  Other reasons  Don’t Know  Refused |
|  | [If other from SEVBFWHYA or SEVBFWHYB] Write a few words summarising the response, in English. | …………………… |
| 7b.2.2 | If households get fewer things for money from the forest in the next ten years, would there be more poverty, less poverty, or no change? | More poverty  No change  Less poverty  Don’t Know  Refused  Less food |
| 7b.2.3 | If households get more things for money from the forest in the next ten years, would there be more poverty, less poverty, or no change? | More poverty  No change  Less poverty  Don’t Know  Refused  Less food |

**Section 7c: Scenario set 3**

|  | **QUESTION** | **RESPONSE** |
| --- | --- | --- |
|  | We have nearly finished our questions. When we ask you these questions, we would like to think about the next ten years. You might not know the answer, but I would like you to think about what could happen. We want to remind you that there is no plan or project coming from this research. Do you understand? | |
| 7c.1.1.a | Do you think there will be a change in who has land over the next ten years? | Yes  No  Don’t Know  Refused |
| 7c.1.1.b | [If yes] Why is this?  [NOTE] Let the respondent answer first. If they don't know, then read out the responses. Select all that apply. | More people sugarcane farming  More people food crop farming  More people growing other cash crops other than sugarcane  Other reasons  Don’t Know  Refused |
| 7c.1.1.c | [If other from SEVCWHYA] Write a few words summarising the response, in English. | …………………… |
| 7c.1.2 | Who will get more land? Would it be households with more money, or households with less money, or others not mentioned would get more land, or do you think there would be no change? | Households with more money  Households with less money  Others  No one  Don’t Know  Refused |
| 7c.1.3 | Who will get less land? Would it be households with more money, or households with less money, or others not mentioned would get more land, or do you think there would be no change? | Households with more money  Households with less money  Others  No one  Don’t Know  Refused |
| 7c.1.4 | Where would this land come from? | Clearing the forest  Buying or renting land from other farmers  Taking land from other farmers  Buying or renting land from others who own land but don’t farm  Taking land from others who own land but don’t farm  Others  Don’t Know  Refused |
| 7c.1.5 | If those households got less land in the next ten years, would there be more poverty, less poverty, or no change in those households? | More poverty  No change  Less poverty  Don’t Know  Refused |
| 7c.1.6 | If those households got less land in the next ten years, would there be more hunger, less hunger, or no change in those households? | More hunger  No change  Less hunger  Don’t Know  Refused |

**Section 7d: Scenario set 4**

|  | **QUESTION** | **RESPONSE** |
| --- | --- | --- |
|  | We have nearly finished our questions. When we ask you these questions, we would like to think about the next ten years. You might not know the answer, but I would like you to think about what could happen. We want to remind you that there is no plan or project coming from this research. Do you understand? | |
| 7d.1.1.a | I want you to imagine that the price of sugarcane increased, and people wanted to grow more sugarcane over the next ten years. When you imagine this, do you think there would be a change in who has land over the next ten years? | Yes  No  Don’t Know  Refused |
| 7d.1.1.b | [If yes] Why is this?  [NOTE] Let the respondent answer first. If they don't know, then read out the responses. Select all that apply. | More people sugarcane farming  More people food crop farming  More people growing other cash crops other than sugarcane  Other reasons  Don’t Know  Refused |
| 7d.1.1.c | [If other from SEVDWHYA] Write a few words summarising the response, in English. | …………………… |
| 7d.1.2 | If the price of sugarcane increased and people wanted to grow more sugarcane, who would get more land?  Would it be households with more money, or households with less money, or others not mentioned would get more land, or do you think there would be no change? | Households with more money  Households with less money  Others  No one  Don’t Know  Refused |
| 7d.1.3 | If the price of sugarcane increased, and people wanted to grow more sugarcane, who would get less land?  Would it be households with more money, or households with less money, or others not mentioned would get less land, or do you think there would be no change? | Households with more money  Households with less money  Others  No one  Don’t Know  Refused |
| 7d.1.4 | Where would this land come from? | Clearing the forest  Buying or renting land from other farmers  Taking land from other farmers  Buying or renting land from others who own land but don’t farm  Taking land from others who own land but don’t farm  Others  Don’t Know  Refused |
| 7d.1.5 | If those households got less land in the next ten years, would there be more poverty, less poverty, or no change in those households? | More poverty  No change  Less poverty  Don’t Know  Refused |
| 7d.1.6 | If those households got less land in the next ten years, would there be more hunger, less hunger, or no change in those households? | More hunger  No change  Less hunger  Don’t Know  Refused |

## Supplementary methods 2: Depression instrument

Total eight-item Patient Health Questionnaire (PHQ-8) scores were not used within the statistical analysis^1,2^. Instead, the PHQ-8 was used to estimate latent depression risk. The dataset was split into training (2/3 of observations) and test (1/3 of observations) sets. Using the training set, parallel analysis using polychoric correlation and a weighted least squares estimator suggested extracting one factor using the ‘psych’ package^3^. Exploratory factor analysis with the training set suggested each item was reasonably well loaded on the one extracted factor, with good model fit using the ‘semTools’ package^4^. Confirmatory factor analysis using the test data yielded similar results, although with a poorer model fit (likely because of the small size of the test dataset). A one-factor graded response model using all data suggested was fit using the ‘mirt’ package^5^. This analysis indicated that the instrument had reasonable discriminatory power, although with some floor and ceiling effects. This model had a root mean square error of approximation (RMSEA) of 0.048 (95% confidence interval 0.033 - 0.065), comparative fit index (CFI) of 0.864, Tucker–Lewis index (TLI) of 0.810, and standardised root mean square residual (SRMR) of 0.044. One set of plausible values were extracted from a graded responses model run with each of the ten imputed datasets. Plausible values were used because the software used in the statistical model could not estimate factors from ordinal data.

“Thinking too much” is a colloquial term used to indicate degrees of psychological distress in the study area^6^. An additional variable corresponding to the format of the PHQ-8 was therefore included, asking respondents if they “think too much”. This variable was not used in the formal assessment of depression risk but was instead used to triangulate the study with the previous qualitative study results (see S12: Supplementary analysis 1).

## Supplementary methods 3: Prior probability details

Table S1. The a priori hypothesised associations between exposure and outcome variables in the structural equation model, the distributional form and type of prior, the supporting evidence, and associated hyperparameters. Key: ‘+’ = positive association; ‘-’ = negative association; ‘?’ = uncertain direction of the association; *N* = normal distribution, and *B* = beta distribution.

| **Outcome variable** | **Expected association** | **Exposure variable** | **Distributional form of the priors** | **Type of prior** | **Prior evidence** | **Picture of plot** | **Hyperparameters** |
| --- | --- | --- | --- | --- | --- | --- | --- |
| Depression | (+) | Food insecurity | Normal | Moderately informative | Food insecurity is a widely recognised and strong social determinant of common mental disorders, including depression^7-10^. Food insecurity also appears to be a prominent stressor associated with psychological distress in the study site and other parts of Uganda^6,11^. | 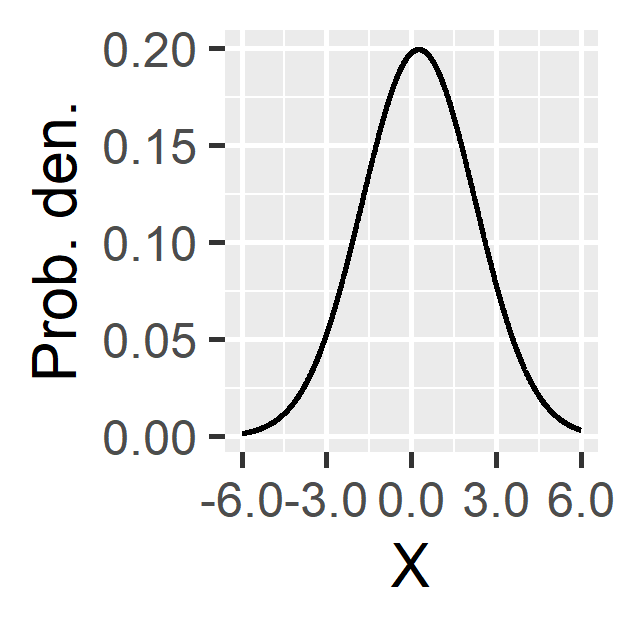 | $N(0.25, 4)$ |
| Depression | (+) | Economic poverty | Normal | Moderately informative | Economic poverty is also a widely recognised and strong social determinant of common mental disorders, such as depression^9,10,12^. Again, economic poverty also appears to be a prominent stressor associated with psychological distress in the study site and Uganda generally^6,13^. | 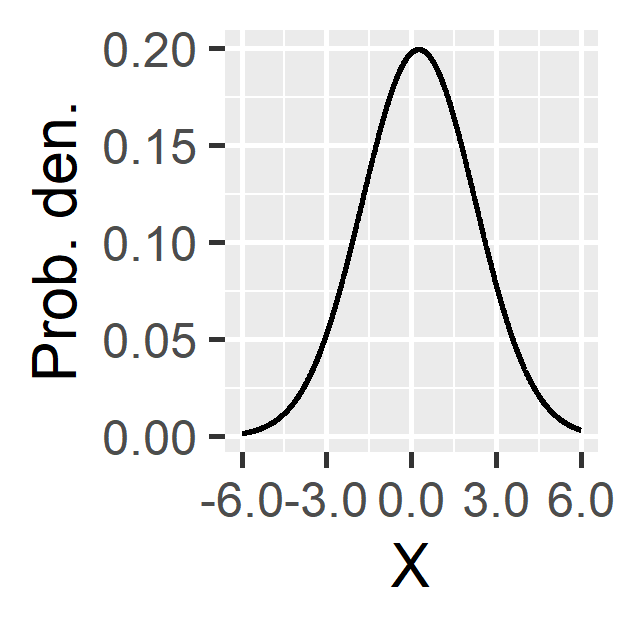 | $N(0.25, 4)$ |
| Food insecurity | (+) co-variance | Forest use | Beta | Moderately informative | Prior evidence from the study site suggests that legally and illegally harvested forest resources are an important source of food and income (used to purchase food) for some households, particularly in times of need^6^. And so, food-insecure households appear more likely to be forest users, but forest use alleviates food insecurity. | 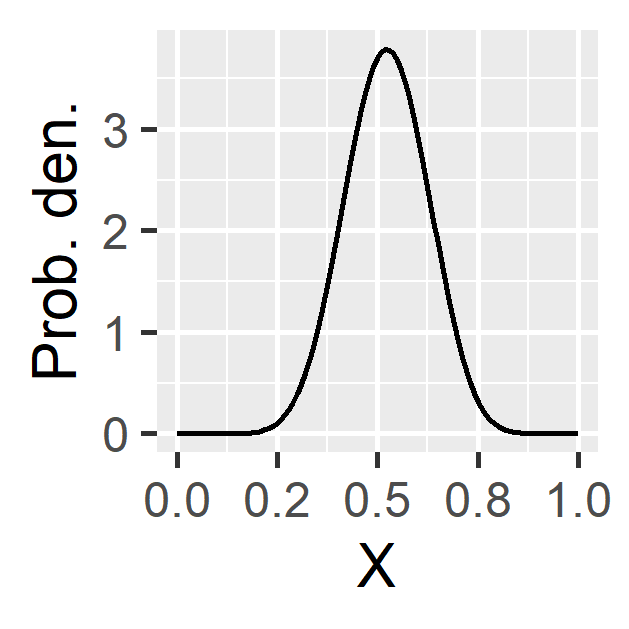 | $B(12, 11)$ |
| Food insecurity | (-) | Farm size | Normal | Moderately informative | Multiple on-farm factors influence food security, including absolute farm size^14,15^. Previous research found that small farm sizes were considered a major cause of food insecurity for residents^6^. | 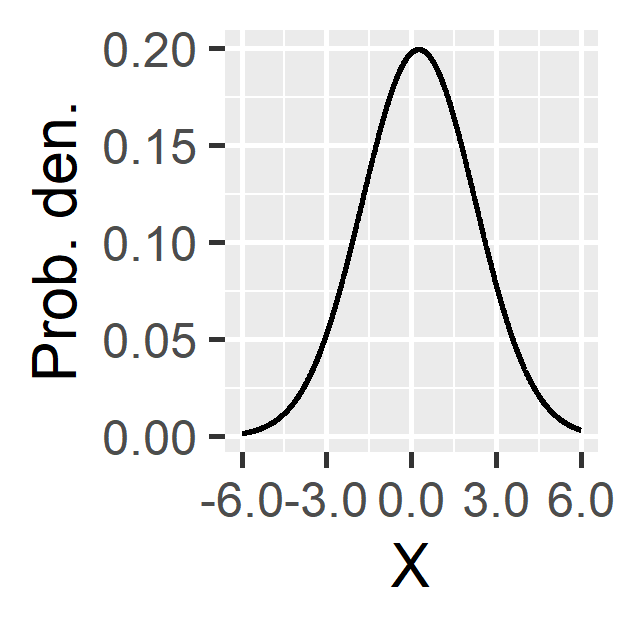 | $N(-0.25, 4)$ |
| Food insecurity | (+) | Economic poverty | Normal | Moderately informative | In general, food insecurity and economic poverty are often closely tied and sometimes treated as sub-dimensions of general poverty (e.g., ^10^). Previous research within the study site suggests a strong association between food insecurity and economic poverty, with income often used to purchase food, especially in times of need^6^. | 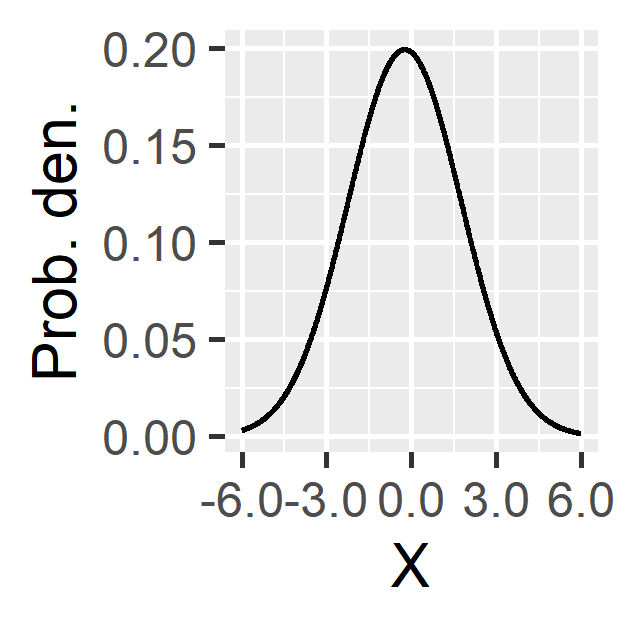 | $N(0.25, 4)$ |
| Food insecurity | (-) | Distance for forest reserve | Normal | Moderately informative | Previous research suggests that crop-raiding may threaten food security in the study area and mostly occurs close to the forest edge^6,16,17^. | 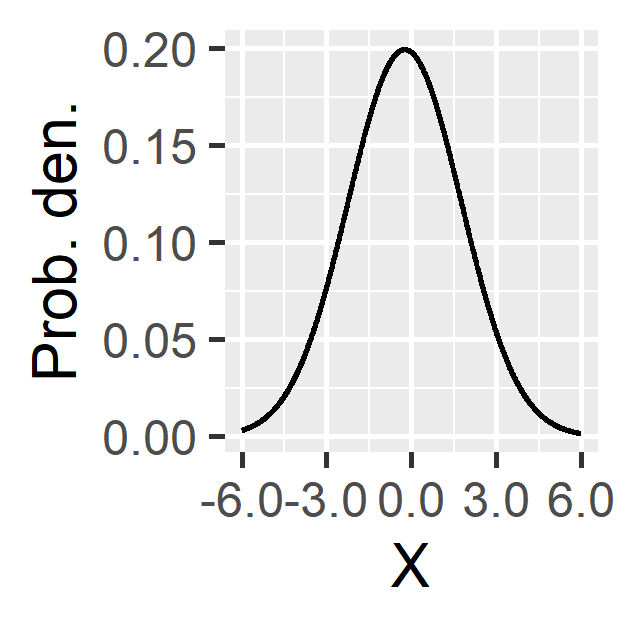 | $N(-0.25, 4)$ |
| Economic poverty | (+) co-variance | Forest use | Beta | Moderately informative | Prior research in the study site suggests that legally and illegally harvested forest resources are to be an important source of income for some households; poor households are more likely to be forest users, but forest resource utilisation helps alleviate poverty^6^. | 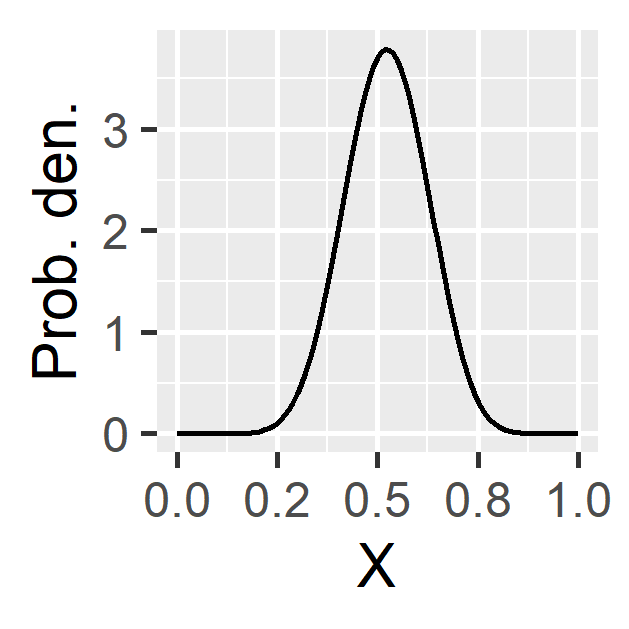 | $B(12, 11)$ |
| Economic poverty | (-) co-variance | Farm size | Beta | Moderately informative | Most land outside the forest reserves within the study site has been cleared for agriculture, meaning there is little available land for agricultural expansion^18^. Residents of the area reported rising land prices leading to increasingly inequitably distributed farm sizes between wealthier and poorer households^6^. | 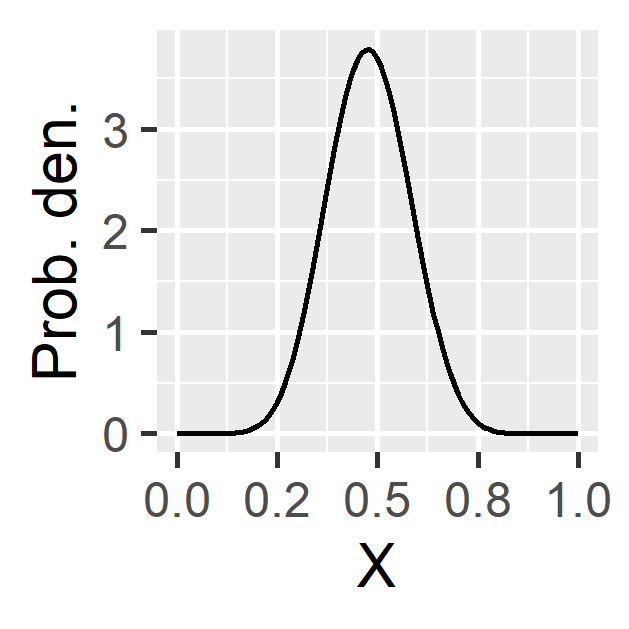 | $B(11,12)$ |
| Depression | (+) | Age | Normal | Moderately informative | Although the association may not be linear, prior evidence suggests a general positive association between age and poor mental health in Uganda^13,19^. | 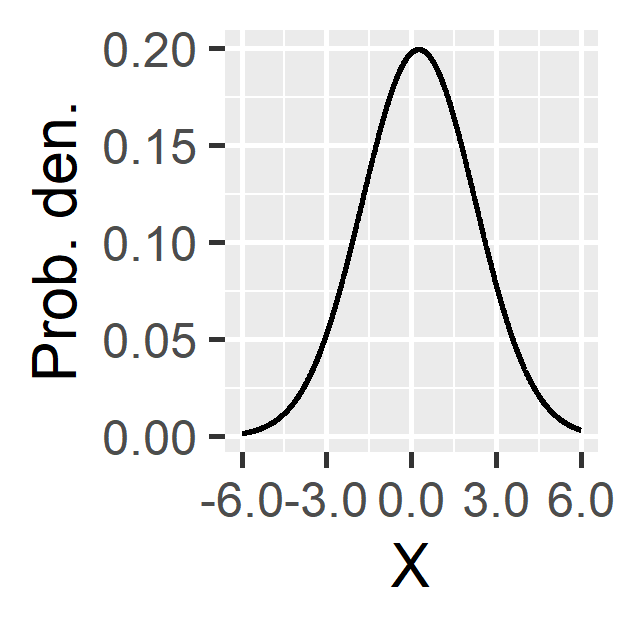 | $N(0.25, 4)$ |
| Depression | (+) | Gender | Normal | Moderately informative | Evidence from Uganda indicates women may be at higher risk of poor mental health than men ^11,13^. Prior research in the case study suggests women might face greater threats to mental health due to having fewer assets and less income, greater childcare responsibilities, and other factors^6^. | 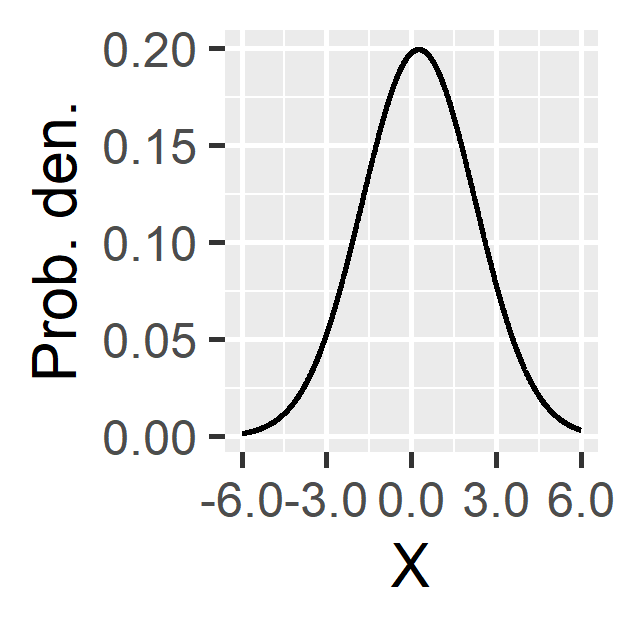 | $N(0.25, 4)$ |
| Depression | (-) | Education | Normal | Moderately informative | While the relationship between educational level and mental health can be complex^9^, educational attainment is often associated with socioeconomic status^10^. Some evidence for Uganda suggests higher levels of education are associated with better mental health^13^. | 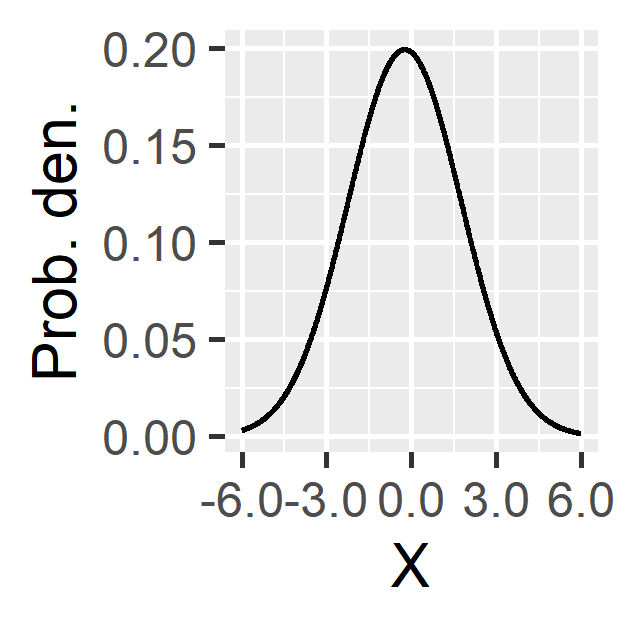 | $N(-0.25, 4)$ |
| Depression | (-) | Social support | Normal | Moderately informative | Significant evidence suggests those with less social support (from spouses, family, and friends) have worse mental health, including research in Uganda^11,20,21^. | 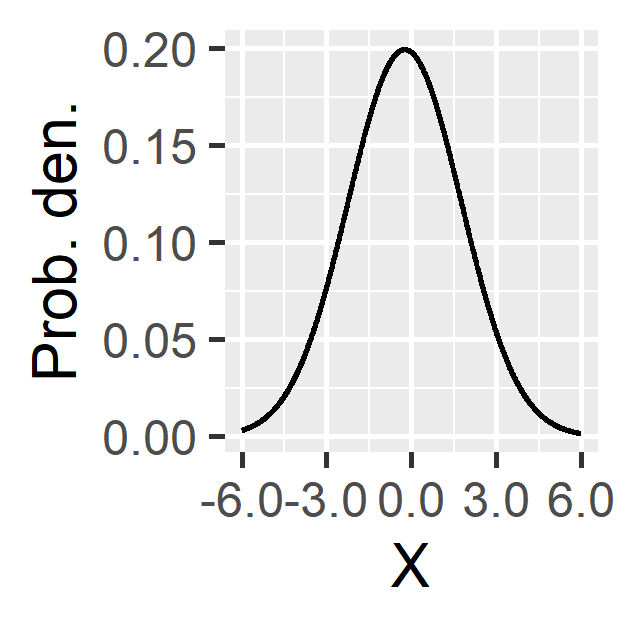 | $N(-0.25, 4)$ |
| Depression | (+/?) | Marital status: divorced or widowed | Normal | Moderately informative | Although married individuals tend to have better mental health, this relationship may be more complex is contexts where polygamy is common^22,23^. Prior evidence from Uganda suggests those who are divorced or widowed are at around double the risk of depression than the general population in Uganda^13^. We adopt a strongly informative prior for ‘divorced or widowed’ but a weakly informative prior for ‘never married’. | 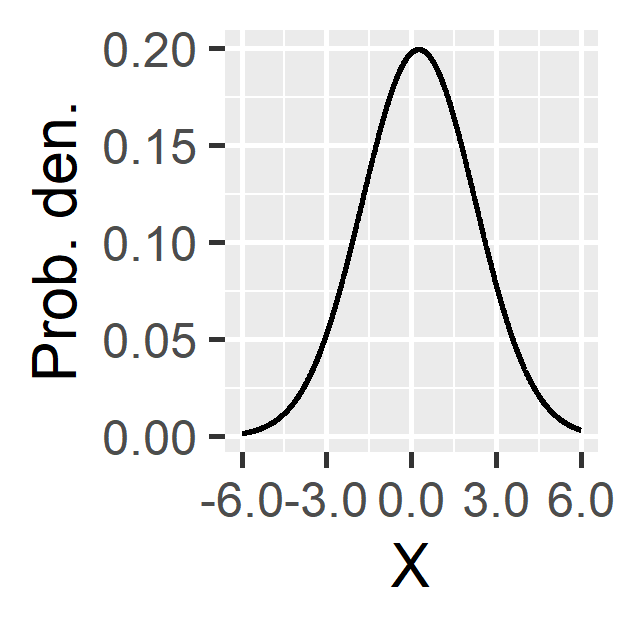 | $N(0.25, 4)$ |
| Depression | (+/?) | Marital status: never married | Normal | Weakly informative |  | 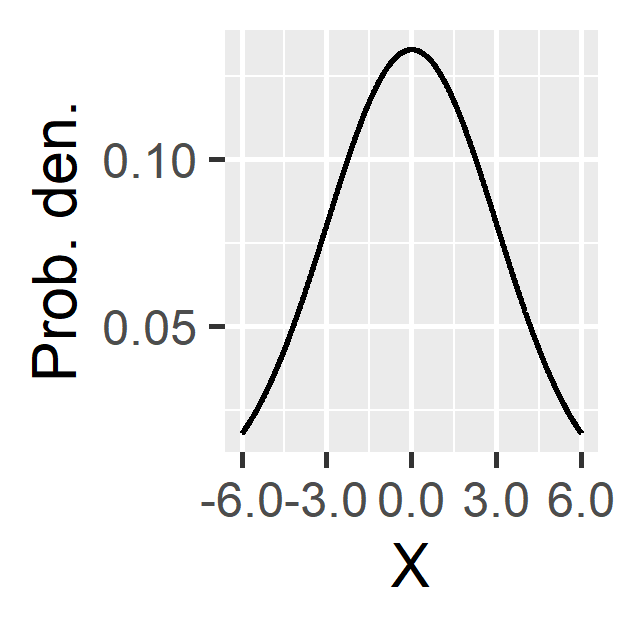 | $N(0, 9)$ |
| Depression | (-) co-variance | General health | Beta | Moderately informative | A large amount of research describes bi-directional co-morbidity between mental and physical health^24^. | 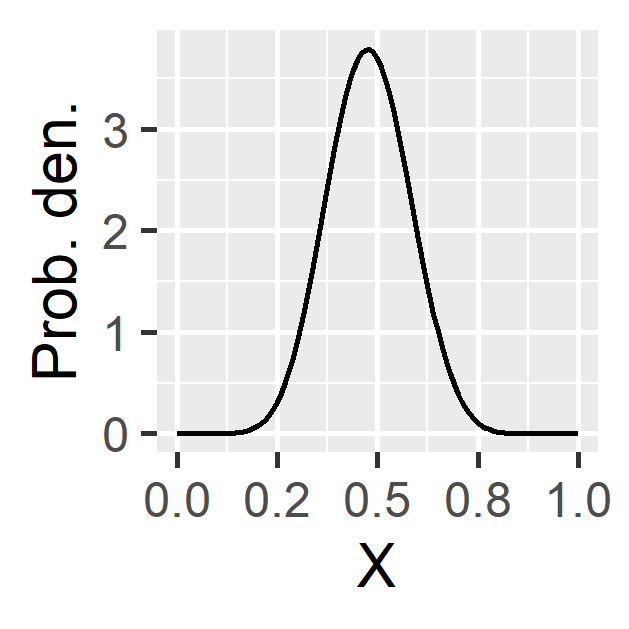 | $B(2, 3)$ |
| Depression | (+) | Alcohol consumption | Normal | Moderately informative | Evidence suggests a positive association between drinking and depression risk, with the former tending to causally influence the latter^25^. | 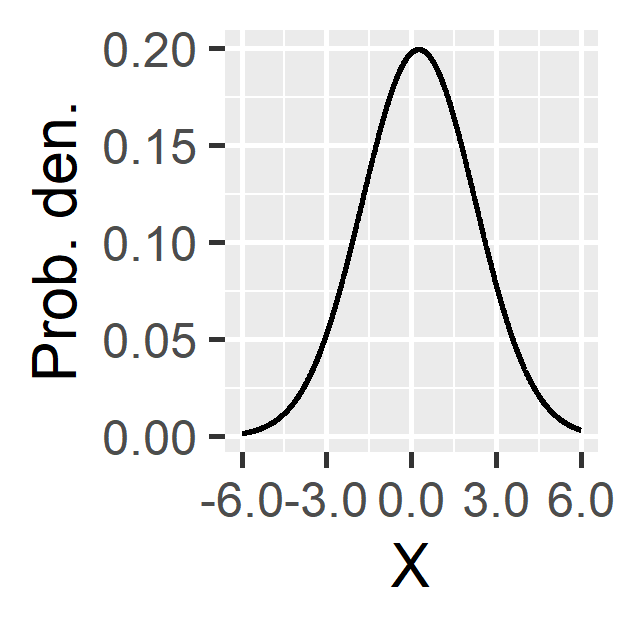 | $N(0.25, 4)$ |
| Depression | (+) | Smoking | Normal | Moderately informative | Tobacco smoking is often positively associated with depression risk, although the direction of causality is uncertain^26^. Some evidence that smoking might be a risk factor in Uganda^19^. | 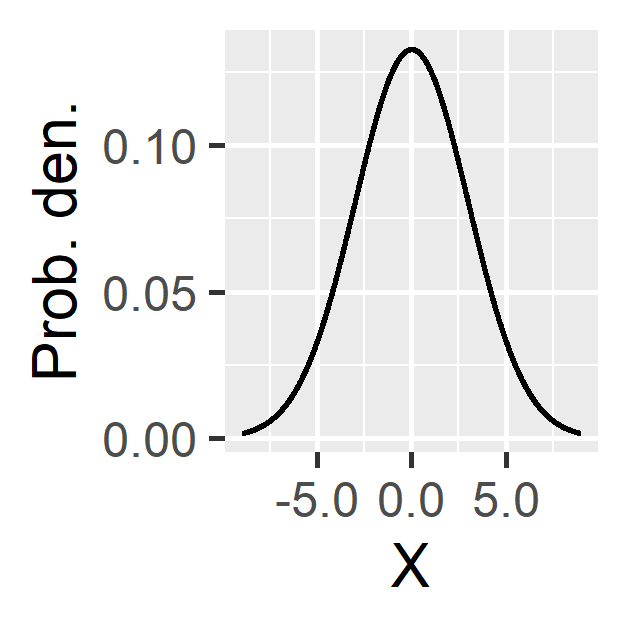 | $N(0.25, 4)$ |
| Depression | (?) | Community name (all) | Normal | Weakly informative | Prior research within the study site suggests community-level differences in access to utilities, markets, and sources of employment^6^. The inclusion of this variable controls for these differences. | 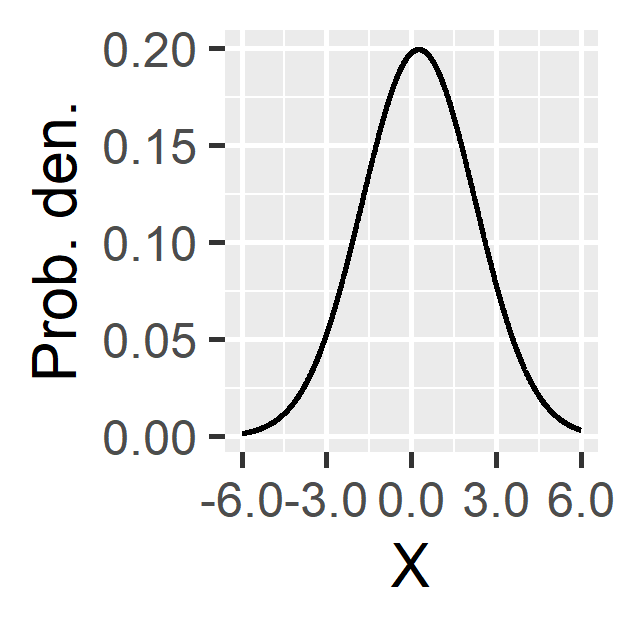 | $N(0,9)$ |

## Supplementary methods 4: FIES instrument

The Food Insecurity Experience Scale (FIES) was used to estimate food insecurity. Rasch, two-parameter, and three-parameter logistic item response models were fit. The two-parameter model had 24.1 lower Akaike information criterion scores than the next best model, and so was chosen. This model had a RMSEA of 0.087 (95% CI 0.073 - 0.102), CFI = 0.971, TLI of 0.959, and SRMR of 0.051. Ten sets of plausible values were extracted, as above.

## Supplementary methods 5: Asset index

A logistic principal component analysis was conducted with 21 household assets (Table S2). The number of principal components to estimate in the model was selected through an iterative process. This process involved repeating the logistic principal component analysis ten times, starting with extracting one component and ending with the extraction of ten. Out of these ten candidate models, we choose the one whose factor scores had the largest linear association with an external measure of subjective financial strain. This measure of financial strain is a single-item question recommended by the National Academy of Medicine^[[1]](#footnote-1),27,28^. This analysis suggested using nine principal components within the model (which explained 85.7% of the total variance). The first component was used as a proxy for economic poverty.

Table S2. Household assets that were include (‘Yes’) or excluded (‘No’) in a logistic principal component analysis to construct an asset index, used as a proxy for economic poverty.

| **Assets** | **Included** |
| --- | --- |
| A gas cooker | **No** |
| At least two sets of clothes for every person in the household | Yes |
| Two goats | **No** |
| At least two meals per day | Yes |
| Mains electricity | **No** |
| A wooden bed (frame) | Yes |
| A big solar panel | Yes |
| At least two Jerry cans | Yes |
| One mattress or more | Yes |
| A television | **No** |
| Brick walls for your house | Yes |
| A motorbike | **No** |
| Two hoes | Yes |
| A blanket for every person in the household | Yes |
| A bank account | **No** |
| A bicycle | Yes |
| A water tank | **No** |
| A pair of shoes for every member of the household | Yes |
| Iron sheets on your roof | Yes |
| A radio | Yes |
| Three saucepans | Yes |
| Solar battery | **No** |
| A mobile phone | Yes |
| Two or more plastic chairs | Yes |
| A car | **No** |
| A fridge | **No** |
| A cement floor for your house | Yes |
| A member in a savings group | Yes |
| A sofa | Yes |
| Two or more wooden chairs | Yes |
| Soap | Yes |

## Supplementary methods 6: Forest instrument

We designed an instrument with seven Likert-scaled items to estimate forest use. Parallel analysis and exploratory factor analysis with the training data were performed, followed by confirmatory factor analysis with the test data. Parallel analysis with the training set suggested the extraction of two factors. Exploratory factor analysis suggested that four items were heavily loaded on the first factor, two items were cross-loaded, and the final item was heavily loaded on the second factor. Therefore, a two-factor graded response model was fit (Table S3). This model had an RMSEA of 0.087 (95% CI 0.068 - 0.106), CFI of 0.966, TLI of 0.941, and SRMR of 0.048. The four items heavily loaded on the first factor specifically related to food and income, while the three items heavily loaded on the second factor related to non-specific forest dependence. Therefore, one set of plausible values associated with the first dimension – deemed most relevant for this study – were extracted from each imputed dataset.

Table S3. Loadings within two-factor graded response model. Ten sets of plausible values of the first factor were extracted and used in the primary analysis.

| **Statement** | **First factor** | **Second factor** |
| --- | --- | --- |
| Your household gets things from the forest that help you a lot. |  | 0.787 |
| Your household gets good money from things in the forest. | 0.860 |  |
| The forest helps you buy things you need, like food, school fees, or medicine. | 0.888 |  |
| It would be very bad for your household if you could not get things from the forest. | 0.261 | 0.638 |
| Your household gets food from the forest. | 0.771 |  |
| Getting things from the forest helps your household to survive. | 0.305 | 0.682 |
| Your household would have no money or food if you could not go to the forest. | 0.744 |  |

## Supplementary methods 7: Land instrument

We designed an instrument with six Likert-scaled items to estimate land size. Again, the same steps described to estimate latent depression symptom severity (S1: Depression instrument) were repeated. This analysis suggested the extraction of one factor, and so a one-factor graded response model was fit. This model had an RMSEA of 0.080 (95% CI 0.059 - 0.103), CFI of 0.970, TLI of 0.951, and SRMR of 0.057. As above, one set of plausible values were extracted from each of the imputed datasets.

## Supplementary methods 8: Social support instrument

Social support was estimated using a modified version of the Multidimensional Scale of Perceived Social Support (MSPSS) ^29-31^. The MSPSS is used to estimate latent social support from three sources; friends, family, and significant others. The modification of the MSPSS involves using six of the 12-items, as indicated by Slavin, et al. ^32^. We further simplified the instrument by reducing the number of response levels from seven to five to simplify. Furthermore, we also altered some of the phrases and changed from the first to the second person following piloting. For instance, one of the original statements is “I can count on my friends when things go wrong”, which we changed to “You know your friends will be around to help when trouble finds you.”

The modified version of the MSPSS was used to estimate latent social support, following steps similar to those described in the main text for estimating latent depression. The parallel analysis with the training data suggested the extraction of two factors. Subsequent exploratory factor analysis using all responses suggested three items were heavily loaded onto the first factor, one item was cross-loaded, and two items were loaded on a second factor (Table S4). This model had an RMSEA of 0.083 (95% CI 0.061 - 0.107), CFI of 0.970, TLI of 0.944, and SRMR of 0.057. The first factor related to social support from family or one’s partner, which was deemed more relevant than support from friends, and therefore ten sets of plausible values associated with the first factor were extracted.

Table S4. Loadings within two-factor graded response model. Ten sets of plausible values of the first factor were extracted and used in the primary analysis.

| **Statement** | **First factor** | **Second factor** |
| --- | --- | --- |
| There is a special person around you when you are in need. | 0.578 |  |
| Your family tries to help you. | 0.864 |  |
| You get the comfort and support you need from your family. | 0.902 |  |
| You know your friends will be around to help when trouble finds you. |  | 0.819 |
| You can talk about your problems with your friends. |  | 0.711 |
| There is a special person with whom you can share joys and sorrows. | 0.327 | 0.440 |

## Supplementary methods 9: Patterns of missing data

Approximately 0.2% of the data were missing (Figure S1). This missingness is partly attributed to one of the questions (indicated by ‘SOCJOY’) mistakenly not being asked in an early version of the survey. Additionally, several respondents did not wish to respond to questions about their land size (e.g., those questions staring with the indicator ‘LAND…’).


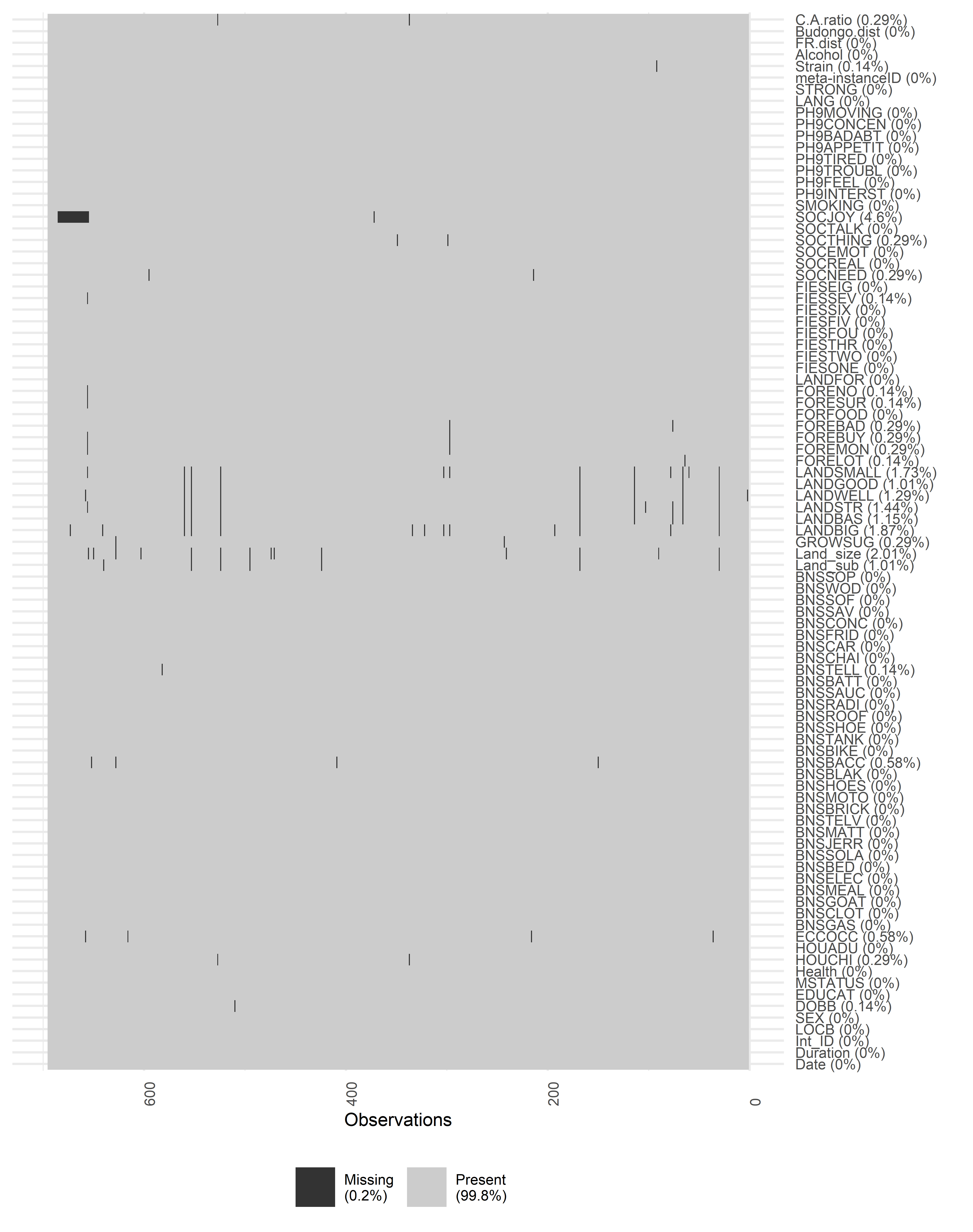


Figure S1. Patterns of missing data within our data.

## Supplementary methods 10: MICE

Missing values were assumed to be missing at random in relation to the response variable and so were substituted with synthetic values through multivariate imputation by chained equations, using the ‘mice’ package^33^. This imputation created ten datasets containing the imputed data. Ordinal variables were imputed using proportional odds models, numeric variables with predictive mean matching, and unordered categorical variables with polytomous logistic regression. The pre- and post-imputation distributions of these variables were consistent.

## Supplementary methods 11: Model diagnostics

The models were evaluated according to the ten steps described in the *When to worry and how to Avoid the Misuse of Bayesian Statistics* (*WAMBS)-Checklist*^34^.

1. “Do you understand the priors?”: TP discussed the priors (shown in S3: Prior probability details) with EJMG and AK.
2. “Does the trace-plot exhibit convergence?”: We inspected the trace-plots, potential scale reduction factor from the Gelman-Rubin Diagnostic, and the Geweke Diagnostic for the model run with the first of the ten imputed datasets. These diagnostics suggested the models had converged, with equality between the first and second half of iterations.
3. “Does convergence remain after doubling the number of iterations?”: The number of pre- and post-burn-in iterations doubled to 8,000 each (16,000 in total), and trace plots, potential scale reduction factor, and Geweke Diagnostic were re-examined. These results suggested that local convergence was not an issue.
4. “Does the histogram have enough information?”: We inspected the histograms of estimates in each iteration, and there appeared to be sufficient information to approximate the posterior, with a single clear peak and sloping sides for each parameter.
5. “Do the chains exhibit a strong degree of autocorrelation?”: We found a small amount of autocorrelation, but it was not accompanied by convergence issues, so it was not concerning.
6. “Does the posterior distribution make substantive sense?”: The posterior distributions were consistent with our hypothesis and a plausible mean and spread.
7. “Do different specifications of the multivariate variance priors influence the results?” We re-run the model with a multivariate variance prior of “gamma(1, 0.05)”, which had no discernible effect on the posterior distribution.
8. “Is there a notable effect of the prior when compared with noninformative priors?”. We repeated the analysis with the software’s default weakly informative priors, with no discernible effect on the posterior distribution.
9. “Are the results stable from a sensitivity analysis?” We re-ran the analysis twice, first increasing all hyperparameters (e.g., shifting from *N*(0.25, 4) to *N*(0.50, 4)), then decreasing them (e.g., shifting from *N*(0.25, 4) to *N*(0.00, 4)). These changes had no discernible effect on the posterior distribution.
10. “Is the Bayesian way of interpreting and reporting model results used?” We presented the median, 95% and 50% credibility intervals of the posterior distribution.

## Supplementary methods 12: Supplementary analysis 1

We modelled the association between “thinking too much” (the outcome variable) and latent depressive symptom severity (using the raw PHQ-8 scores as the exposure variable) within a Bayesian ordinal regression. We used the software’s default weakly informative priors. The models were fitted with a logit link function and were run for 4000 burn-in and 4000 post-burn-in iterations across four Markov chains and using the Markov Chain Monte Carlo sampler. The analysis was repeated with all ten imputed datasets, and the prior distributions were pooled. PHQ-8 scores were strongly positively correlated with “thinking too much” (Table S5).

Table S5. The results of an ordinal logistic regression examining the association between “thinking too much” and Patient Health Questionnaire (PHQ-8) scores on the log-odds scale.

| **Variable** | **Estimate** | **Standard error** | **95% CI** |
| --- | --- | --- | --- |
| Threshold one (Not at all \| Few days) | 1.08 | 1.19 | 0.77-1.53 |
| Threshold two (Few days \| More than half the days) | 9.46 | 1.21 | 6.49-13.89 |
| Threshold three (More than half the days \| Nearly every day) | 19.19 | 1.23 | 12.88-28.83 |
| PHQ-8 score | 1.27 | 1.02 | 1.22-1.31 |

## Supplementary methods 13: Supplementary analysis 2

We repeated the primary analysis but replaced depressive symptom severity with “thinking too much”. Assuming that responses to “thinking too much” were driven by an underlying latent variable, we scaled and centred the variable and treated it as continuous. The same priors, number of iterations and chains, seed, and sampling algorithm as used in the main analysis was also used here.

The results of this analysis were generally consistent with the primary analysis (Figure S2). However, in contrast with the primary analysis, age had a significant positive association with “thinking too much”, but gender and education did not. Further analysis would be needed to understand these differences. However, these results suggest that “thinking too much” and depression may affect slightly different demographic groups.


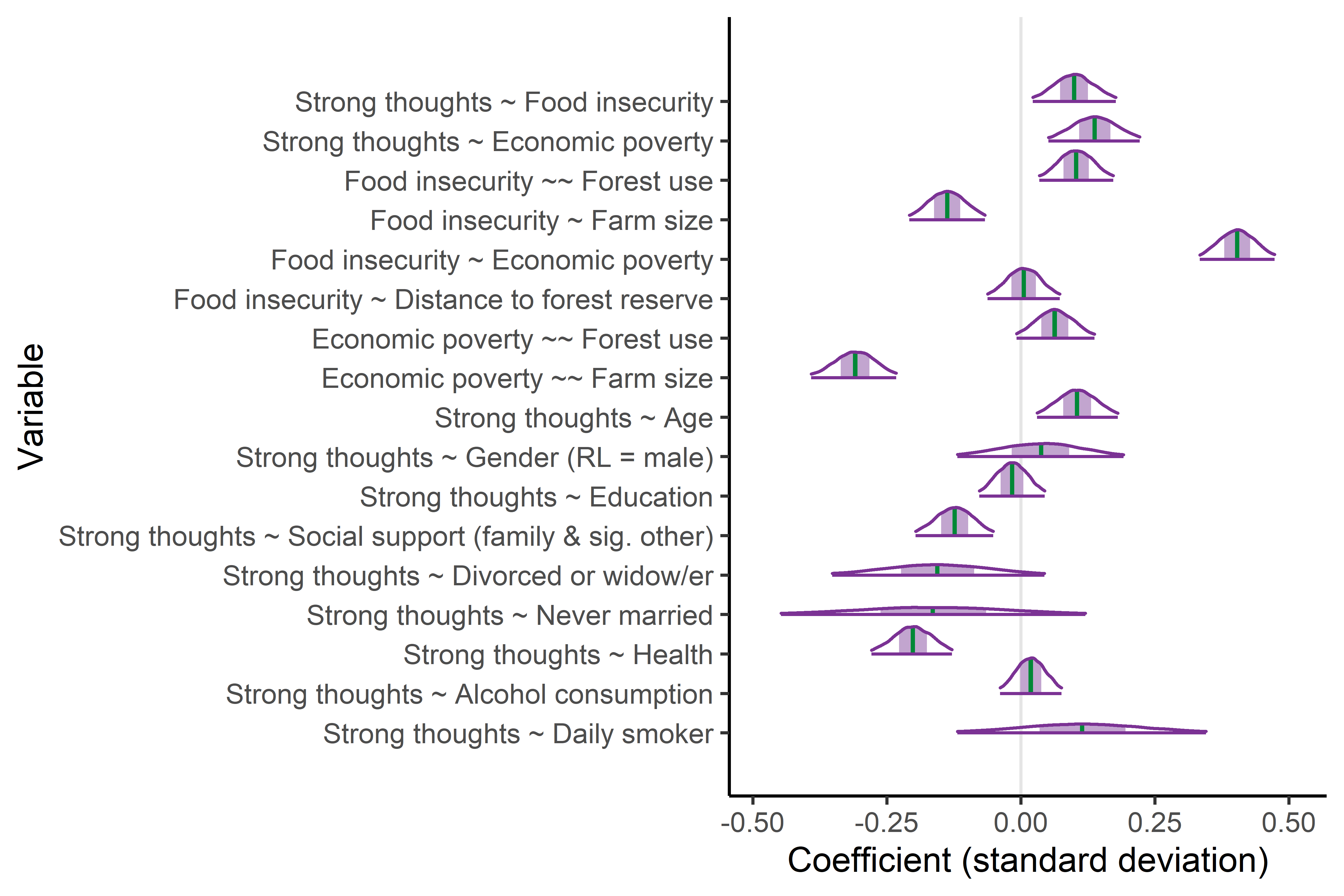


Figure S2. The coefficient estimates from the Bayesian structural equation model using data from 695 respondents. The vertical green line represents the point estimate (median of the posterior distribution), the dark purple line represents the 95% credibility interval, and the shaded area represents the 50% credibility interval. The estimated associations between depressive symptom severity and the community dummy variables are not shown. Coefficient estimates are presented in standard deviations.

**References**

1 Kroenke, K., Spitzer, R. L. & Williams, J. B. W. The PHQ-9: Validity of a brief depression severity measure. *Journal of General Internal Medicine* **16**, 606-613, doi:10.1046/j.1525-1497.2001.016009606.x (2001).

2 Wu, Y. *et al.* Equivalency of the diagnostic accuracy of the PHQ-8 and PHQ-9: A systematic review and individual participant data meta-analysis. *Psychological Medicine* **50**, 1368-1380, doi:<https://doi.org/10.1017/S0033291719001314> (2020).

3 psych: Procedures for psychological, psychometric, and personality research v. 1.9.12 (Northwestern University., Evanston, USA, 2019).

4 semTools: Useful tools for structural equation modeling v. 05-3 (<https://CRAN.R-project.org/package=semTools>, 2020).

5 Chalmers, R. P. mirt: A multidimensional Item Response Theory package for the R environment. *Journal of Statistical Software* **48**, 1-29, doi:10.18637/jss.v048.i06 (2012).

6 Pienkowski, T. *et al.* The role of nature conservation and commercial farming in psychological distress among rural Ugandans. *Land Use Policy* (in review).

7 Weaver, L. J. & Hadley, C. Moving beyond hunger and nutrition: A systematic review of the evidence linking food insecurity and mental health in developing countries. *Ecology of Food and Nutrition* **48**, 263-284, doi:10.1080/03670240903001167 (2009).

8 Jones, A. D. Food insecurity and mental health status: A global analysis of 149 countries. *American Journal of Preventive Medicine* **53**, 264-273, doi:10.1016/j.amepre.2017.04.008 (2017).

9 Lund, C. *et al.* Social determinants of mental disorders and the Sustainable Development Goals: A systematic review of reviews. *The Lancet Psychiatry* **5**, 357-369, doi:10.1016/s2215-0366(18)30060-9 (2018).

10 Lund, C. *et al.* Poverty and common mental disorders in low and middle income countries: A systematic review. *Social Science & Medicine* **71**, 517-528, doi:10.1016/j.socscimed.2010.04.027 (2010).

11 Kinyanda, E. *et al.* Major depressive disorder and suicidality in early HIV infection and its association with risk factors and negative outcomes as seen in semi-urban and rural Uganda. *Journal of Affective Disorders* **212**, 117-127, doi:10.1016/j.jad.2017.01.033 (2017).

12 Patel, V. & Kleinman, A. Poverty and common mental disorders in developing countries. *Bulletin of the World Health Organization* **81**, 609-615 (2003).

13 Kinyanda, E. *et al.* Poverty, life events and the risk for depression in Uganda. *Social Psychiatry and Psychiatric Epidemiology* **46**, 35-44, doi:10.1007/s00127-009-0164-8 (2011).

14 Otsuka, K. Food insecurity, income inequality, and the changing comparative advantage in world agriculture. *Agricultural Economics* **44**, 7-18, doi:<https://doi.org/10.1111/agec.12046> (2013).

15 Fraval, S. *et al.* Food access deficiencies in sub-Saharan Africa: prevalence and implications for agricultural interventions. **3**, doi:10.3389/fsufs.2019.00104 (2019).

16 Tweheyo, M., Hill, C. M. & Obua, J. Patterns of crop raiding by primates around the Budongo Forest Reserve, Uganda. *Wildlife Biology* **11**, 237-247, doi:10.2981/0909-6396(2005)11[237:Pocrbp]2.0.Co;2 (2005).

17 Hill, C. M. Perspectives of “conflict” at the wildlife–agriculture boundary: 10 years on. *Human Dimensions of Wildlife* **20**, 296-301, doi:10.1080/10871209.2015.1004143 (2015).

18 Babweteera, F. *et al.* in *The Ecological Impact of Long-term Changes in Africa’s Rift Valley* (ed A. J. Plumptre) 31-53 (Nova Science Publishing Inc., 2012).

19 Kinyanda, E., Waswa, L., Baisley, K. & Maher, D. Prevalence of severe mental distress and its correlates in a population-based study in rural south-west Uganda. *BMC Psychiatry* **11**, 1-9, doi:10.1186/1471-244X-11-97 (2011).

20 Haber, M. G., Cohen, J. L., Lucas, T. & Baltes, B. B. The relationship between self-reported received and perceived social support: A meta-analytic review. *American Journal of Community Psychology* **39**, 133-144, doi:10.1007/s10464-007-9100-9 (2007).

21 Garipy, G., Honkaniemi, H. & Quesnel-Valle, A. Social support and protection from depression: Systematic review of current findings in western countries. *British Journal of Psychiatry* **209**, 284-293, doi:10.1192/bjp.bp.115.169094 (2016).

22 Bove, R. & Valeggia, C. Polygyny and women's health in sub-Saharan Africa. *Social Science & Medicine* **68**, 21-29, doi:<https://doi.org/10.1016/j.socscimed.2008.09.045> (2009).

23 Coombs, R. H. Marital status and personal well-being: A literature review. *Family Relations* **40**, 97-102, doi:10.2307/585665 (1991).

24 Firth, J. *et al.* The Lancet Psychiatry Commission: A blueprint for protecting physical health in people with mental illness. *The Lancet Psychiatry* **6**, 675-712, doi:10.1016/S2215-0366(19)30132-4 (2019).

25 Boden, J. M. & Fergusson, D. M. Alcohol and depression. *Addiction* **106**, 906-914, doi:10.1111/j.1360-0443.2010.03351.x (2011).

26 Fluharty, M., Taylor, A. E., Grabski, M. & Munaf. The association of cigarette smoking with depression and anxiety: A systematic review. *Nicotine and Tobacco Research* **19**, 3-13, doi:10.1093/ntr/ntw140 (2017).

27 National Academy of Medicine. in *Capturing Social and Behavioral Domains and Measures in Electronic Health Records: Phase 2* (ed Institute of Medicine (US). Committee on the Recommended Social and Behavioral Domains and Measures for Electronic Health Records) Ch. 4, 127-226 (National Academies Press, 2014).

28 Kahn, J. R. & Pearlin, L. I. Financial strain over the life course and health among older adults. *Journal of Health and Social Behavior* **47**, 17-31, doi:10.1177/002214650604700102 (2006).

29 Zimet, G. D., Powell, S. S., Farley, G. K., Werkman, S. & Berkoff, K. A. Psychometric characteristics of the Multidimensional Scale of Perceived Social Support. *Journal of Personality Assessment* **55**, 610-617, doi:10.1080/00223891.1990.9674095 (1990).

30 Zimet, G. D., Dahlem, N. W., Zimet, S. G., Gordon, K. & Farley, G. K. The multidimensional scale of perceived social support the multidimensional scale of perceived social support. *Journal of Personality Assessment* **52**, 37-41, doi:10.1207/s15327752jpa5201 (2010).

31 Shumaker, S. C., Frazier, S. K., Moser, D. K. & Chung, M. L. Psychometric properties of the Multidimensional Scale of Perceived Social Support in patients with heart failure. *Journal of Nursing Measurement* **25**, 90-102, doi:10.1891/1061-3749.25.1.90 (2017).

32 Slavin, V., Creedy, D. K. & Gamble, J. Single item measure of social supports: Evaluation of construct validity during pregnancy. *Journal of Affective Disorders* **272**, 91-97, doi:<https://doi.org/10.1016/j.jad.2020.03.109> (2020).

33 van Buuren S & K, G.-O. mice: multivariate imputation by chained equations in R. *Journal of Statistical Software* **45**, 1-67, doi:10.18637/jss.v045.i03 (2011).

34 Depaoli, S. & Van de Schoot, R. Improving transparency and replication in Bayesian statistics: The WAMBS-Checklist. *Psychological Methods* **22**, 240-261, doi:10.1037/met0000065 (2017).

1. “In the past month, how hard has it been for you to pay for the things you need the most like food, medicine, or clothing? Was it hard or not hard?” If hard: “Was it very hard, hard, or a bit hard?” [↑](#footnote-ref-1)
